# Systematic overexpression of genes encoded by mycobacteriophage Waterfoul reveals novel inhibitors of mycobacterial growth

**DOI:** 10.1093/g3journal/jkac140

**Published:** 2022-06-21

**Authors:** Danielle Heller, Isabel Amaya, Aleem Mohamed, Ilzat Ali, Dmitri Mavrodi, Padraig Deighan, Viknesh Sivanathan

**Affiliations:** Department of Science Education, Howard Hughes Medical Institute, Chevy Chase, MD 20185, USA; Department of Science Education, Howard Hughes Medical Institute, Chevy Chase, MD 20185, USA; Department of Biology, University of Maryland Baltimore County, Baltimore, MD 21250, USA; Department of Biology, University of Maryland Baltimore County, Baltimore, MD 21250, USA; Center for Molecular & Cellular Biosciences, University of Southern Mississippi, Hattiesburg, MS 39406, USA; Department of Biology, Emmanuel College, Boston, MA 02115, USA; Department of Science Education, Howard Hughes Medical Institute, Chevy Chase, MD 20185, USA

**Keywords:** mycobacteriophage, *Mycobacterium smegmatis*, cytotoxicity

## Abstract

Bacteriophages represent an enormous reservoir of novel genes, many of which are unrelated to existing entries in public databases and cannot be assigned a predicted function. Characterization of these genes can provide important insights into the intricacies of phage–host interactions and may offer new strategies to manipulate bacterial growth and behavior. Overexpression is a useful tool in the study of gene-mediated effects, and we describe here the construction of a plasmid-based overexpression library of a complete set of genes for Waterfoul, a mycobacteriophage closely related to those infecting clinically important strains of *Mycobacterium tuberculosis* and/or *Mycobacterium abscessus*. The arrayed Waterfoul gene library was systematically screened in a plate-based cytotoxicity assay, identifying a diverse set of 32 Waterfoul gene products capable of inhibiting the growth of the host *Mycobacterium smegmatis* and providing a first look at the frequency and distribution of cytotoxic products encoded within a single mycobacteriophage genome. Several of these Waterfoul gene products were observed to confer potent anti-mycobacterial effects, making them interesting candidates for follow-up mechanistic studies.

*In this Mutant Screen Report, Heller et al. report the findings of a genome-wide screen to identify novel mycobacteriophage gene products capable of inhibiting mycobacterial growth. The findings of this study represent the core scientific output of a new research project developed by the Howard Hughes Medical Institute (HHMI) to be conducted within the context of undergraduate science courses. The HHMI Science Education Alliance-Gene-function Exploration by a Network of Emerging Scientists, or SEA-GENES project, engages cohorts of undergraduate scientists and their faculty instructors, nationwide, in an exploration of bacteriophage genetics. The Genetics Society of America and G3 are partnering with HHMI to disseminate the student-led discoveries of the SEA-GENES course-based research project to further advance our understanding of this largely uncharted system. Read more about SEA-GENES in the accompanying commentary.*

## Introduction

Bacteriophage genomes are replete with genes of unknown function (Hatfull 2015, 2020). Genomes of ∼3,500 phages isolated on actinobacterial species collectively encode ∼23,000 different “phamilies” of related gene products, of which ∼75% cannot be assigned a predicted function (Cresawn *et al.* 2011; Pope *et al.* 2015; Russell and Hatfull 2017; Hatfull 2018, 2020). Genes encoding products required for core viral functions such as virion structure and assembly or lysis and lysogeny can typically be identified by sequence and synteny; however, due to the remarkable diversity of the population, even these core functions cannot always be recognized and remain largely uncharacterized for most phages (Hatfull and Hendrix 2011; Hatfull 2018, 2020). Phage genomes also encode a variable number of noncore genes, and these are overwhelmingly genes with no known function (NKF) (Hatfull 2015). Typically, NKF genes are small, expressed over the course of the phage life cycle, and though many are dispensable for the lytic growth or lysogeny, they are thought to confer advantage under specific conditions (Hatfull and Hendrix 2011; Dedrick *et al.* 2013, 2017; Pope *et al.* 2014). Indeed, studies probing the functions of these NKF genes continue to expand our understanding of the dynamics and regulation of bacterial infection by phage and reveal novel phage–host interactions that can be exploited for antimicrobial drug development (Liu *et al.* 2004; Rybniker *et al.* 2010, 2011; Kiro *et al.* 2013; Molshanski-Mor *et al.* 2014; Dedrick *et al.* 2017; Ko and Hatfull 2018, 2020; Gentile *et al.* 2019; Montgomery *et al.* 2019).

Gene overexpression is a powerful strategy for studying gene function, linking genes to observable phenotypes. In particular, observation of the effects of phage gene overexpression on bacterial growth has been used as a first indicator to identify phage proteins targeting fundamental cellular processes including replication, protein synthesis, transcription, cell division, and morphology (Liu *et al.* 2004; Rybniker *et al.* 2010, 2011; Kiro *et al.* 2013; Molshanski-Mor *et al.* 2014; Ko and Hatfull 2018, 2020). For example, overexpression of NKF gene *52* from mycobacteriophage Fruitloop was observed to cause a severe growth defect in the host bacterium*, Mycobacterium smegmatis*, with subsequent work demonstrating that gp52 interacts with the essential cell elongation regulator, Wag31 (Ko and Hatfull 2018). A recently published screen of 193 select nonstructural genes from several mycobacteriophages found that 23% were capable of inhibiting growth of the host *M. smegmatis*, demonstrating that cytotoxic genes are abundant within the phage population (Ko and Hatfull 2020). However, as few studies have examined the comprehensive set of genes within a given phage genome, the full abundance and arrangement of these cytotoxic genes within individual phage genomes remain largely unexplored.

**Fig. 1.**
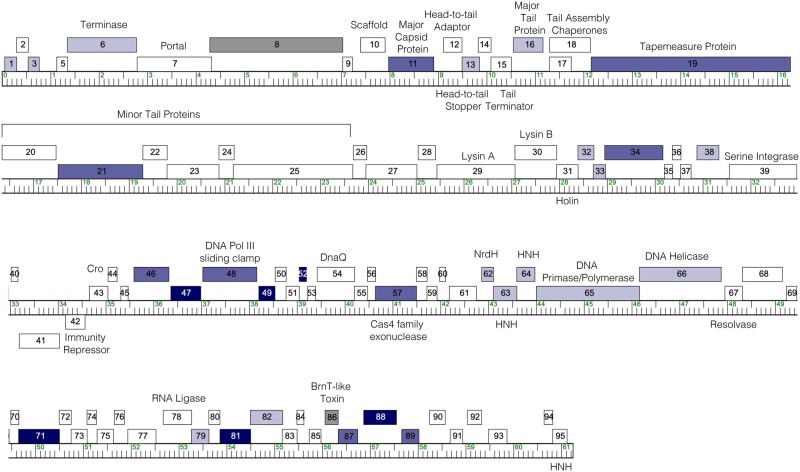
The genome of phage Waterfoul. The Waterfoul genome is shown as a ruler with kbp markers and genes represented by boxes—those above the line are transcribed rightwards and those below are transcribed leftwards. Numbers inside the box correspond to gene numbers and predicted functions are indicated above each gene. Box shading corresponds to cytotoxicity scoring, with white boxes designating genes found to have no effect on *M. smegmatis* growth, gray boxes indicating genes for which transformants could not be recovered (+++*; genes *8* and* 86*), and blue representing observed toxicity in our assay. The saturation of blue boxes corresponds to the severity of growth inhibition using the following scores: light blue (+; reduction in colony size; genes *1, 3, 6, 13, 16, 32, 33, 38, 62, 63, 64, 65, 66, 79,* and* 82*), medium blue (++; 1–3 log reduction in viability; genes *11, 19, 21, 34, 46, 48, 57, 87,* and* 89*), and dark blue (+++; >3-log reduction in viability; genes *47, 49, 52, 71, 81,* and* 88*).

Here, we report the results of a genome-wide overexpression screen of the complete set of genes encoded by a single mycobacteriophage. Phage Waterfoul is a temperate siphovirus isolated on *M. smegmatis mc^2^155* (Jackson *et al.* 2016). Based on gene content similarity, Waterfoul is part of phage cluster K (Russell and Hatfull 2017), which includes several members that have been shown to infect clinically important strains of mycobacteria (Pope *et al.* 2011; Jacobs-Sera *et al.* 2012; Dedrick *et al.* 2019a, 2021; Guerrero-Bustamante *et al.* 2021). Waterfoul encodes 94 predicted protein-coding genes and a single predicted tRNA(Trp) (Fig. 1), and as is typical for phage genomes, most genes (∼60%) have no assigned functions (Jackson *et al.* 2016; Russell and Hatfull 2017). In our study, a diverse set of 32 Waterfoul genes were found to be cytotoxic when expressed from a multi-copy plasmid in the host bacterium *M. smegmatis*, including 17 NKF genes and several genes with predicted core viral functions. This systematic screen reveals that a significant fraction of the genes encoded within a single phage genome (34%) are capable of impairing bacterial growth, and that cytotoxic genes are distributed throughout the genome, often occurring in clusters.

## Materials and methods

### Growth of mycobacteria and mycobacteriophage

*M.* *smegmatis* mc^2^155 was grown at 37°C in Middlebrook 7H9 (Difco) broth supplemented with 10% AD (2% w/v Dextrose, 145 mM NaCl, 5% w/v Albumin Fraction V), 0.05% Tween80, and 10 µg/ml cycloheximide (CHX) or on Middlebrook 7H10 (Difco) agar supplemented with 10% AD and 10 µg/ml CHX. For the transformation of *M. smegmatis* mc^2^155, electrocompetent cells were electroporated with ∼50–100 ng of pExTra plasmid DNA, recovered in 7H9 broth for 2 h at 37 °C with shaking, and transformants selected on 7H10 agar supplemented with 20 µg/ml Kanamycin (GoldBio). Colonies were typically visible after 3 days at 37°C and used in cytotoxicity assays after 4–5 days of incubation. Waterfoul was propagated on *M. smegmatis* mc^2^155 grown at 37°C in the presence of 1 mM CaCl_2_ and no Tween in Middlebrook media and top agar.

### Construction of the pExTra Waterfoul library

The pExTra plasmid was derived from vector pST-KT, a gift from Vinay Nandicoori (Addgene plasmid # 44561; http://n2t.net/addgene: 44561; RRID: Addgene_44561) (Parikh *et al.* 2013). To generate the empty vector plasmid pExTra01, the pST-KT plasmid was amplified by PCR (NEB Q5 HotStart 2× Master Mix) with primers oDH117 and oDH153 to remove the encoded His_6_ and Flag tags. This linear PCR product was then assembled with a second DNA fragment by isothermal assembly (NEB 2× HiFi Master Mix). This second fragment, amplified from plasmid pTNDs-mcherry (a generous gift from Graham Hatfull) using primers oDH152 and oDH154, harbors the *mcherry* coding sequence downstream of an RBS-containing sequence (5′-AAGCTTGATCCGATAACACAGGAACAGATCT) adopted from plasmid pJEM15 (Timm *et al.* 1994; Ko and Hatfull 2018).

Each Waterfoul gene was PCR-amplified (NEB Q5 HotStart 2× Master Mix) from a high-titer Waterfoul lysate using a forward primer complementary to the first 15–25 bp of the gene sequence, introducing a uniform ATG start codon, and a reverse primer complementary to the last 15–25 bp of the gene sequence, including the native stop codon (Supplementary Table S1). All forward primers contained a uniform, RBS-containing 5′ 21-bp sequence and all reverse primers contained a separate 5′ 25-bp sequence; these added sequences are a perfect match to the pExTra plasmid flanking the site of insertion. Linearized pExTra plasmid was prepared via PCR (NEB Q5 HotStart 2× Master Mix) of pExTra01 using divergent primers pExTra_F and pExTra_R and assembled with each gene insert by isothermal assembly (NEB HiFi 2× Master Mix). Recombinant plasmids were recovered by the transformation of *Escherichia* *coli NEB5α F’I^Q^* and selection on LB agar supplemented with 50 µg/ml Kanamycin. Fruitloop *52* wild-type and mutant inserts were cloned into pExTra using a similar strategy, with inserts amplified from pTNDS-Fruitloop*52* plasmids provided by Graham Hatfull (Ko and Hatfull 2018).

The inserted genes for all recovered pExTra plasmids were sequence-verified using sequencing primers pExTra_uniR and pExTra_seqF; longer genes were also sequenced with internal sequencing primers listed in Supplementary Table S1. Two deviations from the genome sequence reported in GenBank were found in genes *61* and *81*, resulting in substitution C163R in gp81 and a 48-bp in-frame deletion removing residues 147–162 in gp61. These same mutations were found in multiple clones and may represent sequence drift of our Waterfoul lysate. Site-directed mutagenesis was performed to clone the sequences reported in GenBank, and both wild-type and mutant alleles were tested in the plate-based cytotoxicity assay with no difference in the behavior of the alleles observed. Representative results for the sequences matching the GenBank report are shown in Supplementary Fig. S1.

### Cytotoxicity screen and phenotype scoring

For cytotoxicity assays, transformed colonies were resuspended and serially diluted in 7H9 broth then spotted on 7H10 Kan plates supplemented with 0, 1, 10, or 100 ng/ml anhydrotetracycline (aTc; Alfa Aesar). Each strain was tested in triplicate alongside pExTra-Fruitloop52 wild-type and mutant (I70S) controls, and growth was monitored over 5 days at 37 °C. Typically, spot growth was visible after 2–3 days incubation, with effects on colony size and color more apparent after 4–5 days incubation. Cytotoxic phenotypes were scored by comparing the spot dilution out to which cells grew in the presence vs the absence of aTc inducer and classified as either having no effect, being moderately cytotoxic with a 1–3 log reduction in cell viability (++), or being highly cytotoxic, causing complete or near complete (>3-log) inhibition of growth (+++). Strains were also evaluated for aTc-dependent size reduction in individual colonies (+) as compared to the Fruitloop *52-I70S* negative control strain on the same aTc plate and the same strain on plates without inducer. All reported cytotoxic genes were found to cause growth inhibition in 2 or more independent experiments, and there was strong agreement between triplicate samples within each experiment. We do note that the magnitude of toxic effects could vary some between experiments, likely due to slight variations in media or growth conditions; variability was more pronounced in those strains observed to have milder toxic effects, perhaps suggesting that these effects are more concentration-dependent and susceptible to growth fluctuations. The inclusion of the Fruitloop *52* control strains on each experimental plate aided in the evaluation of relative gene-mediated effects.

### Waterfoul genomic analysis

The Waterfoul genome map was created using the web-based tool Phamerator (phamerator.org) (Cresawn *et al.* 2011). Reported gene functions are based on those available in the Waterfoul GenBank record (Accession KX585251). In several cases, these GenBank annotations were supplemented by comparison of functional calls for other phamily members on PhagesDB (Russell and Hatfull 2017), especially those from more recently annotated cluster K mycobacteriophage genomes; these designations were confirmed using protein prediction tools HHPRED (PDB_mmCIF70_12_Oct, SCOPe70_2.07, Pfam-A_v35, NCBI_Conserved_Domains(CD)_v3.18) (Gabler *et al.* 2020), NCBI CDD (Lu *et al.* 2020), and NPS Helix-Turn-Helix predictor (https://npsa-prabi.ibcp.fr/). Transmembrane domains for gp31, 32, 41, 45, 58, and 71 were predicted by TOPCONS (Tsirigos *et al.* 2015) and confirmed by TMHMM (Krogh *et al.* 2001) and SOSUI (Hirokawa *et al.* 1998); gp19 transmembrane domain was predicted by both TMHMM and SOSUI, but not TOPCONS.

## Results and discussion

### A system for studying gene-overexpression-mediated effects on *M. smegmatis* growth

**Fig. 2.**
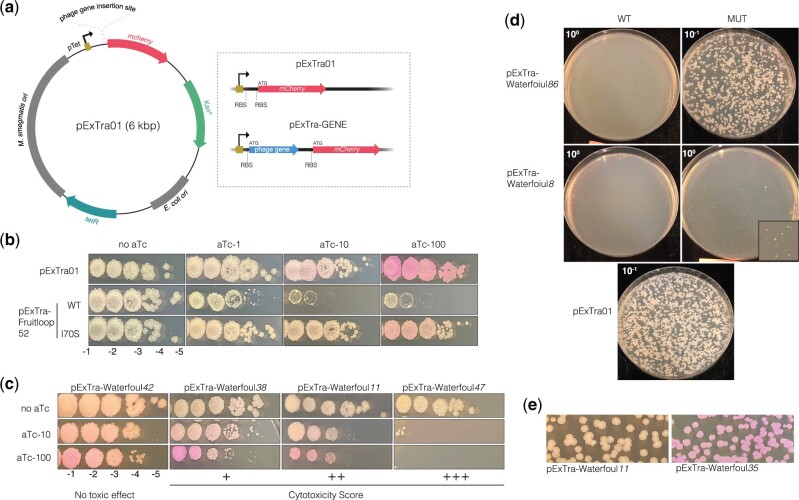
Expression of phage genes from the pExTra plasmid. a) A map of the pExTra01 shuttle vector is shown with the dashed panel highlighting the *pTet*-controlled gene arrangement in empty vector plasmid pExTra01 and in recombinant pExTra phage gene plasmids. b) Spot dilutions of *M. smegmatis* mc^2^155 transformed with the pExTra01 plasmid or two pExTra derivatives expressing wild type or mutant Fruitloop *52* were spotted on 7H10 Kan media containing 0, 1, 10, or 100 ng/ml aTc. c) Results of representative cytotoxicity assays are shown to demonstrate the range of observed growth defects. d) *M. smegmatis* mc^2^155 was transformed with 100 ng of pExTra plasmids encoding either wild-type or nonsense alleles of Waterfoul *8* and *86* and efficiency of transformant recovery compared by plating serial dilutions on 7H10 Kan. e) Examples of typical *M. smegmatis/*pExTra transformants are represented in the top panel (i.e. pExTra-Waterfoul*11*) as compared to those showing aTc-independent *mcherry* expression (i.e. pExTra-Waterfoul*35*).

A plasmid-based expression system was developed to assess the effects of phage gene overexpression in *M. smegmatis*. pExTra, standing for Expression with Transcriptional reporter, is a multi-copy shuttle plasmid with a tetracycline-inducible promoter (*pTet*) driving the expression of an inserted gene and a transcriptionally linked *mcherry* reporter gene, each associated with its own translation initiation signals (Fig. 2a). When transformed with the empty vector plasmid (pExTra01) encoding *mcherry* only, colonies of *M. smegmatis* mc^2^155 turn pink in the presence of the inducer anhydrotetracycline (aTc), with stronger color observed with increasing concentration of aTc, confirming tunable expression from *pTet* (Fig. 2b) (Ehrt *et al.* 2005; Parikh *et al.* 2013). Importantly, no notable growth defects were observed, even at the highest level of induction (Fig. 2b).

To determine the utility of this overexpression system for assessing gene-mediated effects on mycobacterial growth, pExTra derivatives containing alleles of toxic gene Fruitloop *52* between *pTet* and *mcherry* were engineered and tested in a plate-based bacterial growth assay. *M. smegmatis mc^2^155* was transformed with pExTra encoding either wild-type gp52, which was previously shown to inhibit mycobacterial growth upon overexpression, or gp52-I70S, a variant that can be produced at a comparable level as the wild-type protein with minimal effect on mycobacterial growth (Ko and Hatfull 2018). Individual colonies of transformed cells were resuspended, serially diluted, and dilutions plated on 7H10 media with increasing levels of inducer. Consistent with previously published findings, cells expressing wild-type Fruitloop *52* exhibited a pronounced aTc-dependent growth defect (a ∼2-log reduction in growth), whereas those expressing the I70S mutant allele grew to a similar extent in the presence or absence of inducer (Fig. 2b). The pink colony color of cells expressing the mutant provides visible confirmation of *mcherry* transcription and translation from the *pTet* operon, increasing confidence that the linked gene is being expressed.

Thus, the pExTra system enables the controlled expression of phage genes in the host *M. smegmatis*, with the *mcherry* transcriptional reporter providing a visual proxy for gene expression without requiring direct alteration of the gene product by translational fusion. Moreover, the semiquantitative nature of the spot-dilution assay allows for the observation of a range of growth defects, from milder reduction in colony size (e.g. wild-type Fruitloop *52* at 1 ng/ml aTc, Fig. 2b) to multi-log defects in colony number (e.g. wild-type Fruitloop *52* at 10 and 100 ng/ml aTc, Fig. 2b).

Based on these results, we generated an arrayed library for the complete set of Waterfoul protein-coding genes, with each full-length sequence cloned between *pTet* and *mcherry* in pExTra. Each plasmid from the arrayed library of Waterfoul genes was sequence-verified and then used to transform electrocompetent cells of *M. smegmatis* mc^2^155. Three representative transformed colonies for each gene in the library were tested in our plate-based cytotoxicity assay alongside positive and negative control strains expressing the Fruitloop *52* wild-type and the I70S mutant allele, respectively.

### Waterfoul-encoded inhibitors of mycobacterial growth

In total, 30 Waterfoul genes were observed to reproducibly impair *M. smegmatis* growth in an aTc-dependent manner (Table 1, Fig. 1, and Supplementary Fig. S1). Of these, 15 resulted in pronounced growth defects, with 9 resulting in a roughly 1–3-log reduction in colony number (indicated as ++) and 6 with more than a 3-log decrease in colony number (indicated as +++), as compared to when uninduced; an example of each score is provided in Fig. 2c. This frequency of genes with pronounced cytotoxicity upon overexpression (15/92 genes; 16%) is comparable to that observed for a genome-wide cytotoxicity screen for bacteriophage 77 of *Staphylococcus aureus* (16%) (Liu *et al.* 2004). The remaining 15 cytotoxic genes identified in our study resulted in only mild defects in colony size (indicated as +).

**Table 1.** Waterfoul genes observed to inhibit mycobacterial growth.

Gene	Phamily^*a*^	Length (aa)	Predicted function and features	Cytotoxicity^*b*^
*1*	54760	81	NKF	+
*3*	97870	76	NKF	+
*6*	96961	475	Large terminase subunit	+
*11*	15199	310	Major capsid protein	++
*13*	98409	120	Head-to-tail stopper	+
*16*	98249	203	Major tail protein	+
*19*	95098	1,369	Tapemeasure protein; TMD^*c*^ and putative peptidase domain^*d*^	++
*21*	83500	590	Minor tail protein	++
*32*	98277	111	NKF; TMD; DUF2746	+
*33*	97963	84	NKF	+
*34*	55599	405	NKF	++
*38*	95994	152	NKF; HTH DNA-binding	+
*46*	51764	243	NKF	++
*47*	98101	208	NKF; DUF3310	+++
*48*	42207	376	DNA Pol III sliding clamp	++
*49*	8075	112	NKF; DUF1523	+++
*52*	17346	50	NKF	+++
*57*	56834	286	Cas4 family exonuclease	++
*62*	96417	82	NrdH-like glutaredoxin	+
*63*	98588	163	HNH endonuclease	+
*64*	15677	126	HNH endonuclease	+
*65*	14830	719	DNA primase/polymerase	+
*66*	15073	569	DNA helicase	+
*71*	56483	284	NKF; TMD; HTH DNA-binding	+++
*79*	84553	121	NKF	+
*81*	9413	213	NKF	+++
*82*	55054	225	NKF	+
*87*	15142	135	NKF	++
*88*	52229	228	NKF	+++
*89*	56167	119	NKF	++

a Phamily numbers were obtained from phagesdb.org as of January 27, 2022.

b Growth of strains on media supplemented with 10 or 100 ng/ml aTc was compared to the same strains plated on media without aTc. Those denoted as + exhibited aTc-dependent differences in colony size, those denoted as ++ demonstrated a 1–3-log difference in growth in the presence of aTc, and those denoted as +++ demonstrated a severe >3 log reduction in growth in the presence of aTc.

c TMD, transmembrane domain.

d This domain was previously reported in Pedulla *et al.* (2003).

Within this set of 30 cytotoxic genes, 14 have putative functional assignments, including 4 genes encoding products with putative nuclease domains—a Cas4 family exonuclease (gp57), the large terminase subunit (gp6), and 2 HNH-endonucleases (gp63 and gp64)—as well as 3 predicted replisome components—DNA Pol III sliding clamp protein (gp48), DNA primase/polymerase (gp65) and helicase (gp66). The observed cytotoxicity of the former may be a consequence of nonspecific nuclease activity upon overexpression (Rao and Black 1988; Bhattacharyya and Rao 1994), whereas the cytotoxicity of the latter category is consistent with replisome function being both essential and sensitive to the stoichiometry of its constituent parts (Reyes-Lamothe *et al.* 2010; Brüning *et al.* 2016; Homiski *et al.* 2021). We note that the predicted DnaQ-like DNA Pol III subunit (gp54) was not observed to be cytotoxic; this is consistent with the observation that overexpression of *dnaQ* in *Escherichia coli* is not cytotoxic but instead minimizes the accumulation of mutations during the SOS response (Jonczyk *et al.* 1988).

Unexpected cytotoxicity was observed for multiple gene products involved in virion structure and assembly (Table 1). Mild impacts on host growth were seen with overproduction of the major tail protein (gp16) and the putative head-to-tail stopper protein (gp13), whereas a greater impact on viability was observed with production of major capsid protein gp11, one of several minor tail proteins (gp21), and the tape measure protein (gp19). Interestingly, gp19 contains a short, conserved motif previously reported as related to predicted actinobacterial protease proteins (Pedulla *et al.* 2003) that may be responsible for the observed cytotoxicity. Finally, overexpression of the putative NrdH-like glutaredoxin (*62*) was also observed to cause a mild growth defect in *M. smegmatis.*

The remaining 16 growth inhibitory gene products (53%) identified in our study are encoded by genes with no assigned function, including the 6 gene products that resulted in >3-log decrease in viability (+++ genes *47*, *49*, *52*, *71*, *81*, and *88* in Table 1). A subset of these cytotoxic NKF gene products harbor conserved domains, including 2 gene products, gp38 and gp71, with predicted helix-turn-helix DNA-binding motifs; gp71 also contains 4 putative transmembrane helices toward its N-terminus. gp47 contains a conserved domain of unknown function (DUF3310) widespread in phage and bacterial proteomes and previously implicated in nucleotide kinase activity for the T7 protein gp1.7 (Tran *et al.* 2008, 2012, 2014). gp32, encoded by the gene immediately downstream of the putative holin, has a predicted transmembrane segment at its N-terminus followed by a conserved domain of unknown function (DUF2746). Interestingly, gp32 homologues are present in several mycobacteriophage clusters (Russell and Hatfull 2017), and overproduction of the gp32 homologue from cluster F phage Ms6 was previously reported to be toxic in *E. coli*, though cytotoxicity was not tested in the mycobacterial host (Catalão *et al.* 2011).

To date, only a small fraction of the genes encoded by mycobacteriophages have been examined for their impact on mycobacterial growth (Rybniker *et al.* 2008, 2010; Payne and Hatfull 2012; Mehla *et al.* 2017; Ko and Hatfull 2018, 2020; Singh *et al.* 2019). The cytotoxic effects reported here add many novel entries to a growing list of known phage-encoded mycobacterial growth inhibitors. Among these are several potent growth inhibitors that are conserved in phages capable of infecting clinically important mycobacterial strains; this includes gp47, homologs of which are found in phages capable of infecting strains of *Mycobacterium* *tuberculosis*, and gp71 and gp88, both of which are conserved in phages infecting clinical strains of *Mycobacterium* *abscessus* (Jacobs-Sera *et al.* 2012; Dedrick *et al.* 2019a, 2021; Guerrero-Bustamante *et al.* 2021). As with any overexpression screen, it is possible that some of the effects reported here may be artifacts of overexpression. However, given the growing need for new anti-bacterial therapeutics, further characterization of these diverse gene products and the host processes they target, is warranted.

### Waterfoul genes that do not inhibit mycobacterial growth upon overexpression

Of the Waterfoul genes tested for cytotoxicity, 67% caused no appreciable reduction in bacterial growth in our assay, with comparable viability and colony size in the presence or absence of aTc inducer (Fig. 2 and Supplementary Fig. S1). For the large majority of the genes found to be nontoxic to the host (53/62), pink colony color was observed at the higher concentration of aTc, indicating transcription and translation are occurring through the *pTet* operon (Supplementary Fig. S1). However, we cannot rule out that some of these nontoxic gene products may not be soluble or accumulate within cells in our assay.

Among the nontoxic genes, 21 have predicted functions, including several genes for which we would not predict toxicity, for example, the putative immunity repressor (*42*), a gene predicted to be expressed in a lysogen based on our general understanding of repressor function and supported by published expression data for the homologous gene in the cluster K phage ZoeJ (Dedrick *et al.* 2019b). Overproduction of the Waterfoul lysin A (gp29) and lysin B (gp30) proteins alone had no effect on host viability, consistent with reports that these must work in concert with each other and holin to coordinate disruption of the cell envelope (Payne *et al.* 2009; Catalão and Pimentel 2018). Interestingly, a few mycobacteriophage lysin A proteins have been shown to cause holin-independent cell death when expressed in the cytoplasm (Payne and Hatfull 2012), including the Waterfoul homolog from mycobacteriophage Kostya, which was found to cause modest lysis after prolonged induction in liquid (Payne and Hatfull 2012). We did not observe any evidence of clearing in a culture of *M. smegmatis* expressing Waterfoul *29* after ∼16 h of induction with 100 ng/ml aTc (data not shown). It should be noted that this strain is one for which pink color was not observed (on plates or in liquid), and differences in expression level may account for the different behavior of the homologs. Further characterization and comparison of these 2 homologs, which share 66% amino acid identity, could offer interesting insights into the genetic determinants of holin-independent killing. Overproduction of the putative Waterfoul holin protein (gp31) was also found to have no obvious effect on host viability, though this strain displayed robust *mcherry* expression in our assay (Supplementary Fig. S1). While there are reports in the literature of cytotoxicity associated with overexpression of some holin proteins (Catalão *et al.* 2011), it is not known how broadly this applies to the diverse population of holin-like proteins.

### Additional observations of Waterfoul gene expression

Not included in the discussion above are results for Waterfoul genes *8* and *86*. Despite multiple transformation attempts using 2 independently constructed pExTra clones of each plasmid, we were unable to obtain *M. smegmatis* transformants containing pExTra encoding genes *8* or *86*. One potential explanation for this poor transformant recovery is cytotoxicity associated with leaky protein production in the absence of inducer. Indeed, this seemed especially likely for Waterfoul *86*, which is predicted to encode a BrnT-like ribonuclease toxin from the type II BrnT/BrnA toxin-antitoxin system. To test this possibility, we generated versions of pExTra encoding nonsense alleles of *8* and *86* with a stop codon inserted at the second codon position and compared the ability of the wild-type and mutant alleles to transform *M. smegmatis* mc^2^155. In both cases, the nonsense mutation allowed for the recovery of transformants (Fig. 2d), indicating that leaky expression of *8* and *86* from the *pTet* promoter is responsible for poor transformant recovery. This also suggests that these gene products are cytotoxic to the mycobacterial host (designated as +++* in Fig. 1), bringing the total number of Waterfoul-encoded inhibitors to 32. We additionally note that rescue was incomplete for Waterfoul *8* harboring the nonsense mutation, with residual defects still observed for transformant recovery and colony size as compared to the pExTra01 control plasmid (Fig. 2d). Given that some phage genes are known to harbor intragenic translation signals (Catalão and Pimentel 2018), we speculate that production of truncated products of gp8 could account for these observed results.

Finally, although in most cases transformation of *M. smegmatis* mc^2^155 with pExTra derivatives yielded white colonies in the absence of aTc as expected, 6 consistently yielded uniformly pink transformants, presumably due to aTc-independent *mcherry* expression (Fig. 2e and Supplementary Fig. S1). No mutations were found in the *pTet* promoter region of these plasmids, suggesting that these inserted phage genes may harbor promoter sequences that can be recognized by the host transcriptional machinery.

### Insights into genome-wide patterns of host inhibition

The systematic screening of the complete set of Waterfoul genes allows for several genome-wide observations. First, cytotoxic genes can account for approximately one-third (32/94, 34%) of all genes within a phage genome. Though we do not expect these cytotoxic genes to necessarily be employed by the phage to halt host growth during infection, this figure highlights the numerous ways in which the suite of gene products encoded by a phage can interact with host proteins and perturb essential host processes.

Second, gene cytotoxicity can be difficult to predict. For example, overexpression of many structure and assembly genes known to be highly expressed in the host cell during lytic infection (Dedrick *et al.* 2019b) were found to impair host growth, whereas the presence of a transmembrane domain, which may be expected to destabilize the bacterial membrane when present at high concentrations, did not predict cytotoxicity. Of the 7 Waterfoul proteins predicted to contain transmembrane domains (gp19, gp31, gp32, gp41, gp45, gp58, and gp71), only 3 were observed to inhibit growth (Supplementary Fig. S1); those that were not toxic (gp31, gp41, gp45, and gp58) all exhibited pink color on aTc, suggesting that expression was occurring. Cytotoxic genes are also distributed throughout the genome, and their gene products can range in size, from 50 aa to the largest protein encoded by Waterfoul, the 1,369 aa tape measure protein.

Finally, we observed that cytotoxic genes are typically clustered with other cytotoxic genes (Fig. 1). As it is well-established that genes are arranged within phage and bacterial genomes according to shared function, a cytotoxic cluster may thus represent multiple genes that target a common host process. In the search for anti-bacterial therapeutics, cytotoxic clusters might prove a useful strategy for identifying host vulnerabilities well-suited for drug development.

In conclusion, the observations presented here highlight the value of conducting comprehensive genome-wide cytotoxicity screens. In conjunction with further characterization of these Waterfoul-encoded growth inhibitors, systematic screening of additional phage genomes will undoubtedly uncover many more cytotoxic genes, providing important comparisons to further dissect the determinants of cytotoxicity and to better understand the generalizability and relevance of the genome-wide patterns reported here.

## Data availability

All plasmids and plasmid sequences reported in this study are available upon request.

The authors affirm that all data necessary for confirming the conclusions of this article are represented fully within the article and its tables and figures.

Supplemental material is available at *G3* online.

## Supplementary Material

jkac140_Supplemental_Figure_1

jkac140_Supplemental_Figure_1_Legend

jkac140_Supplemental_Table_1
