# Supplementary material for: Systematic overexpression of genes encoded by mycobacteriophage Waterfoul reveals novel inhibitors of mycobacterial growth
Source: G3 (Bethesda). 2022 Jun 21;12(8):jkac140. doi: 10.1093/g3journal/jkac140 (PMC9339283; doi:10.1093/g3journal/jkac140)

Gene	Score
Waterfoul *1*	+
Waterfoul *2*	-
Waterfoul *3*	+
Waterfoul *5*	-
Waterfoul *6*	+
Waterfoul *7*	-

aTc-0 aTc-10 aTc-100

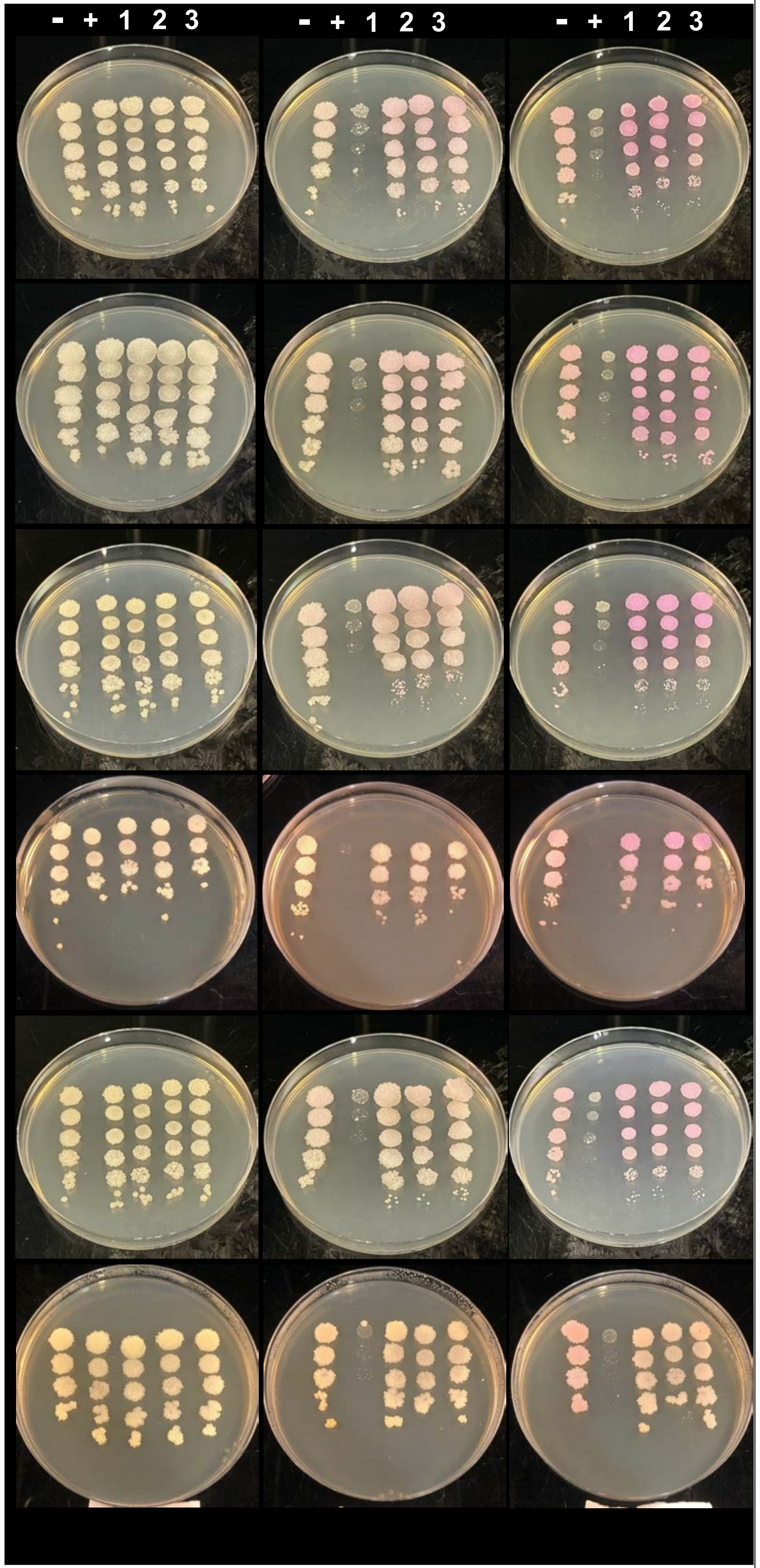

Gene	Score
Waterfoul *9*	-
Waterfoul *10*	-
Waterfoul *11*	++
Waterfoul *12*	-
Waterfoul *13*	+
Waterfoul *14*	-

aTc-0 aTc-10 aTc-100

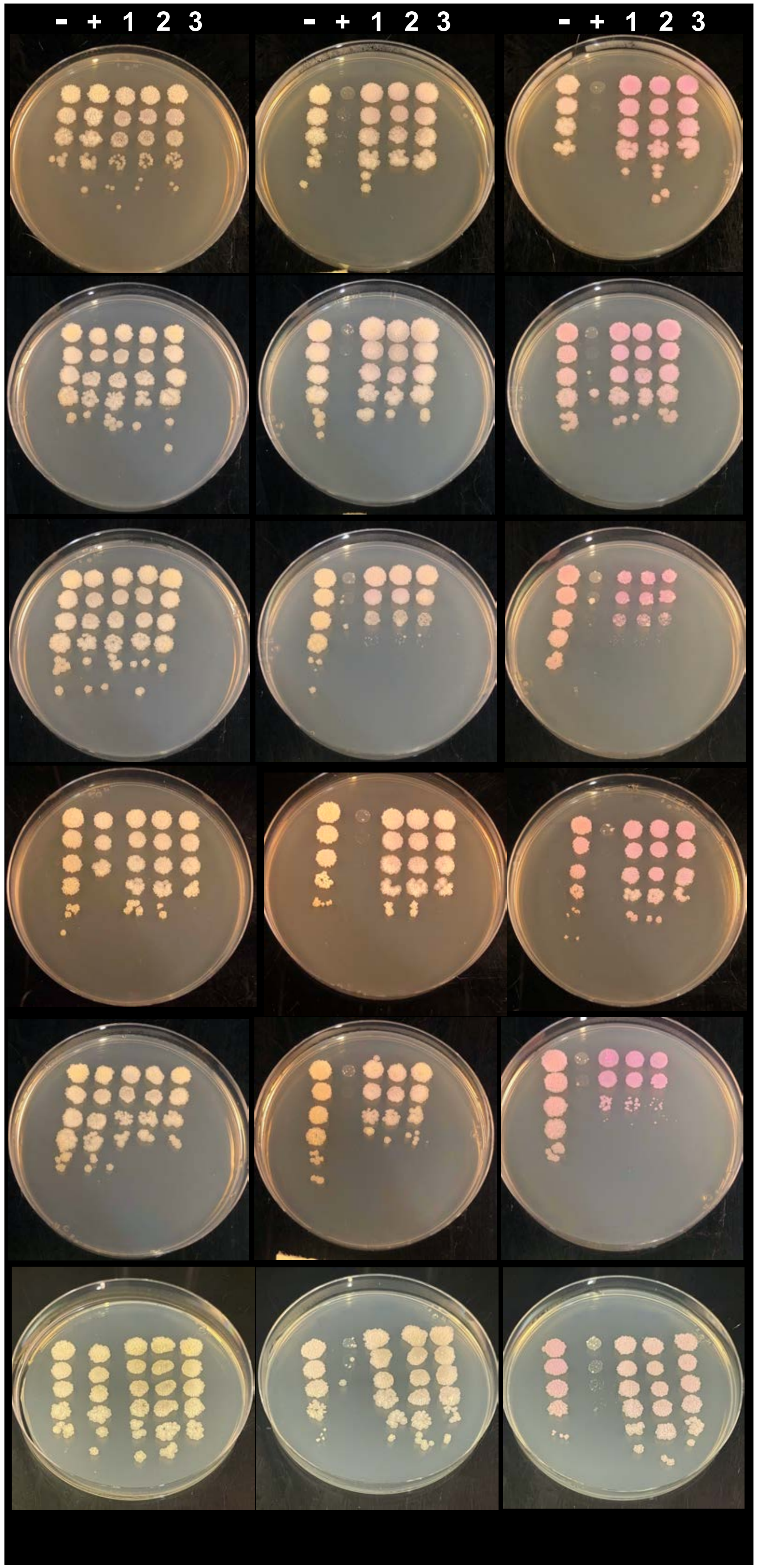

Gene	Score
Waterfoul *15*	-
Waterfoul *16*	+
Waterfoul *17*	-
Waterfoul *18*	-
Waterfoul *19*	++
Waterfoul *20*	-

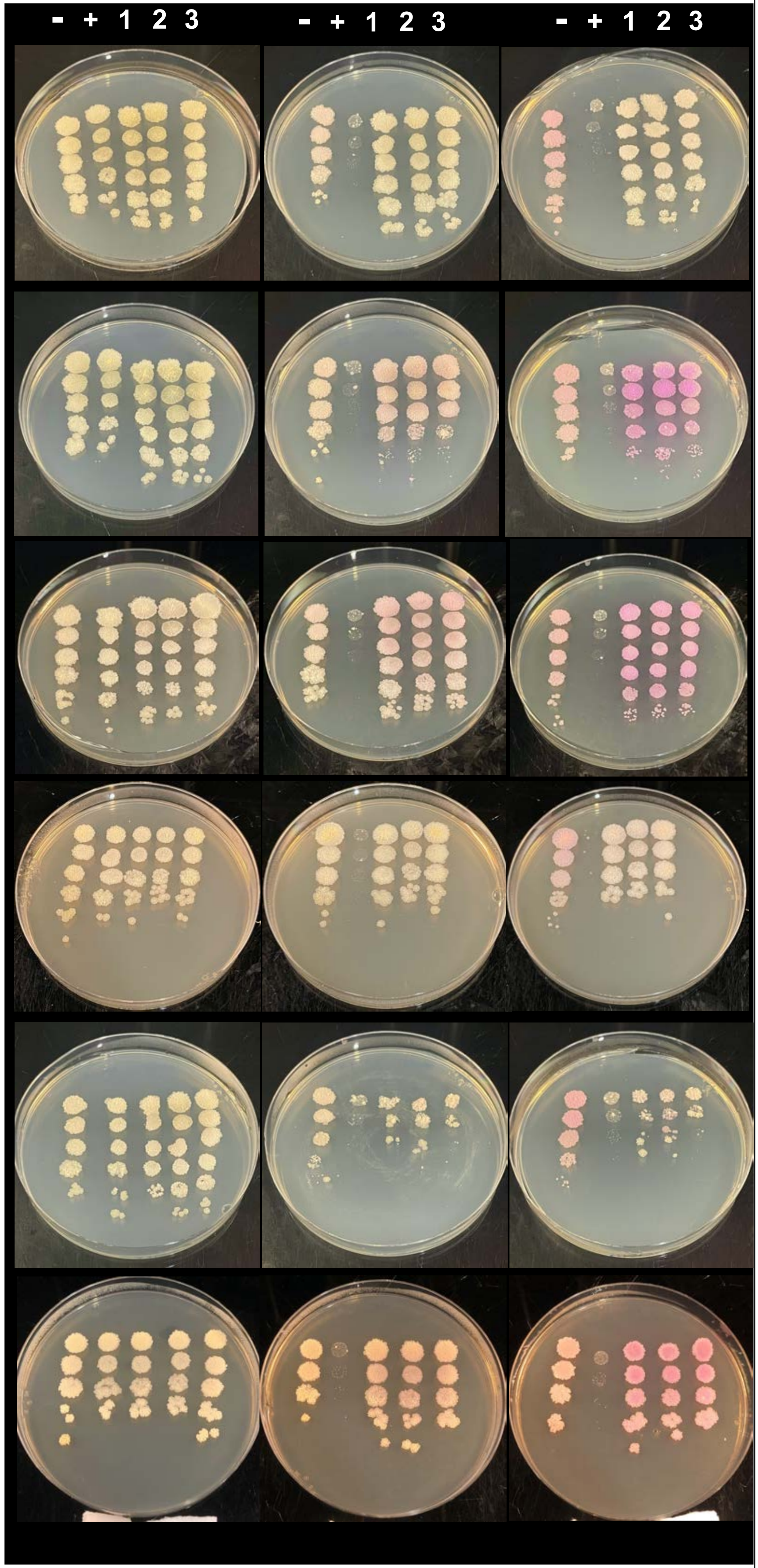

Gene	Score
Waterfoul *21*	++
Waterfoul *22*	-
Waterfoul *23*	-
Waterfoul *24*	-
Waterfoul *25*	-
Waterfoul *26*	-

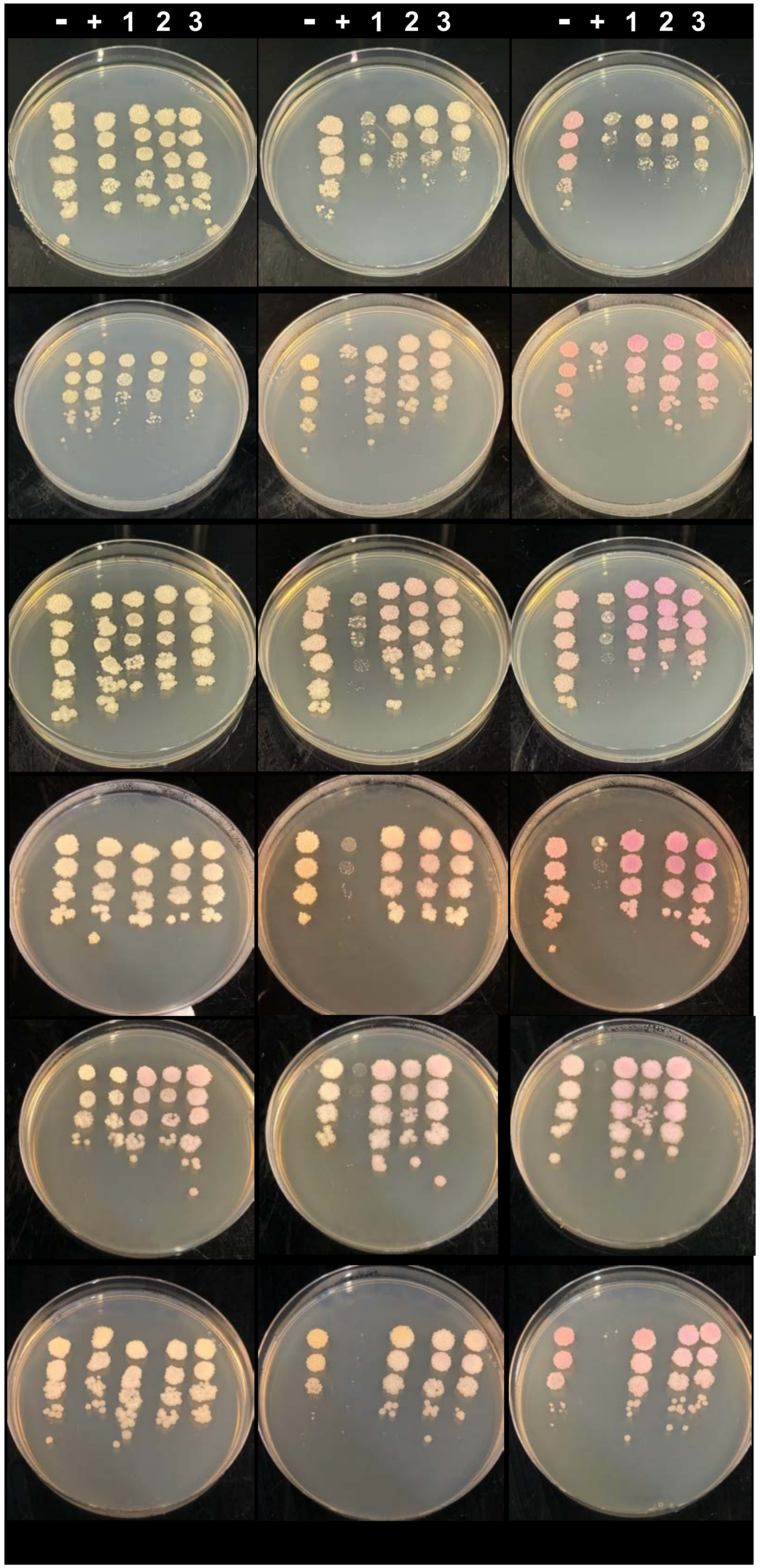

Gene	Score	aTc-0	aTc-10	aTc-100
Waterfoul *27*	-			
Waterfoul *28*	-			
Waterfoul *29*	-			
Waterfoul *30*	-			
Waterfoul *31*	-			
Waterfoul *32*	+			

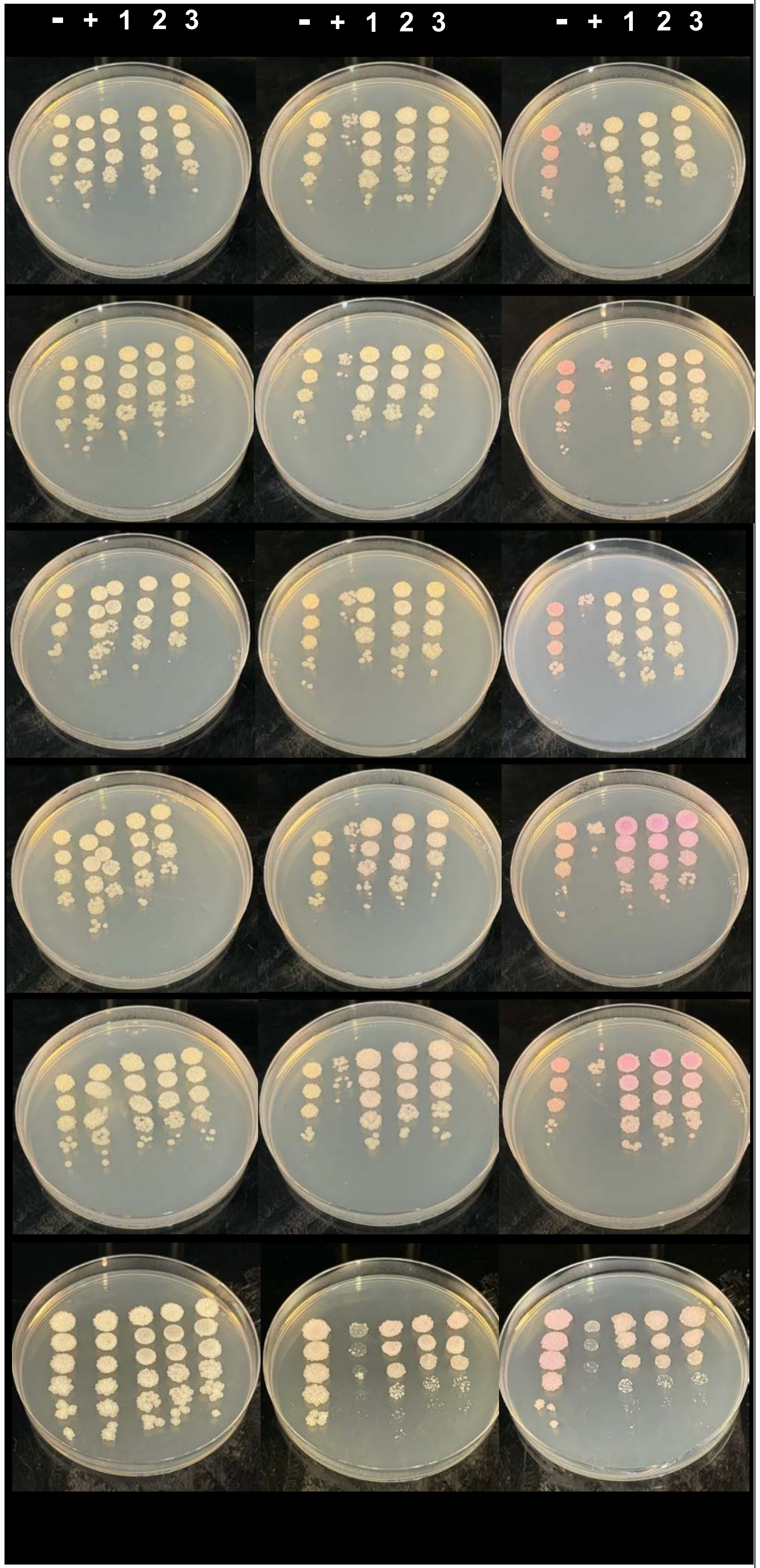

Gene	Score	aTc-0	aTc-10	aTc-100
Waterfoul *33*	+			
Waterfoul *34*	++			
Waterfoul *35*	-			
Waterfoul *36*	-			
Waterfoul *37*	-			
Waterfoul *38*	+			

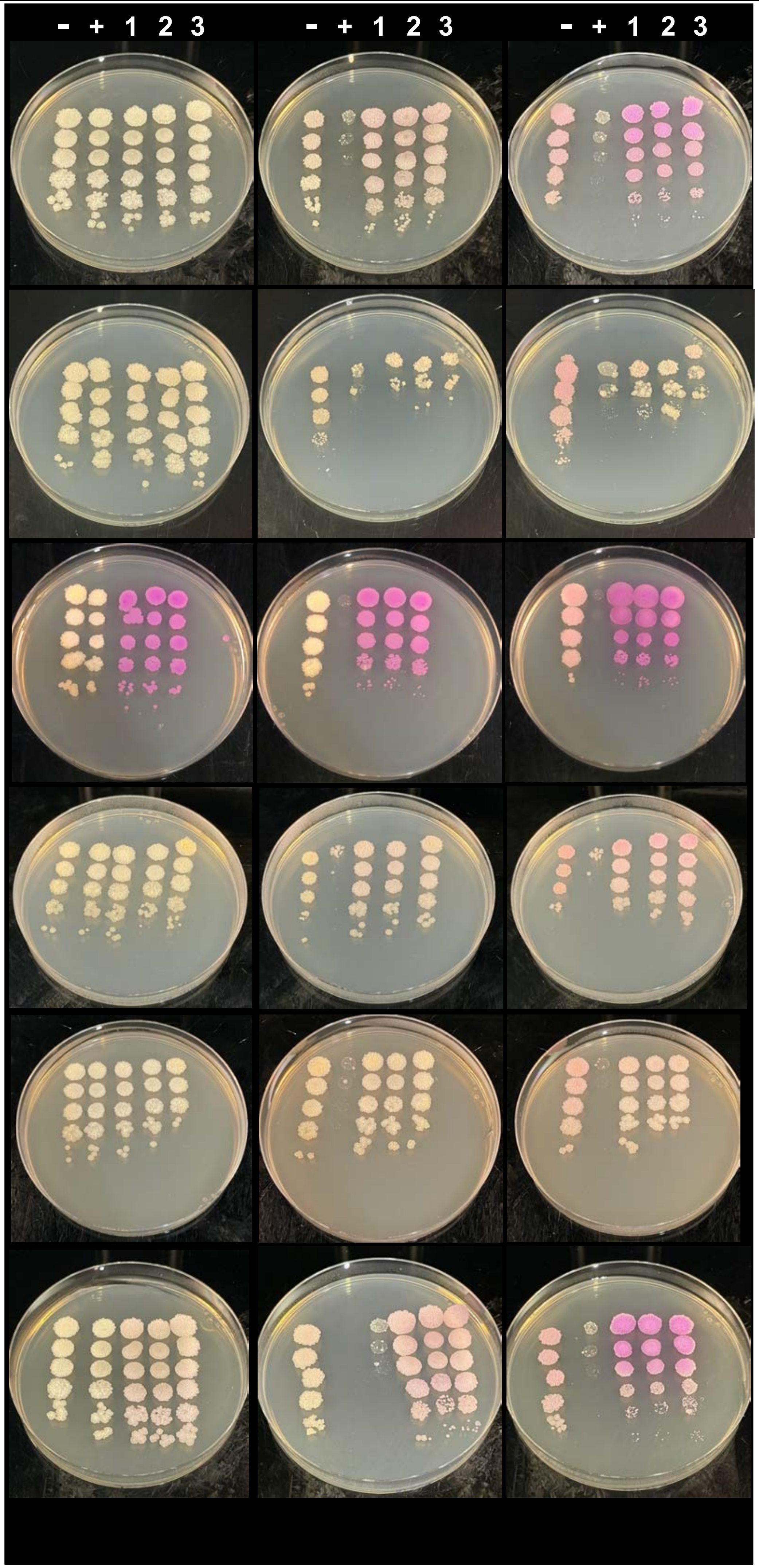

Gene	Score	aTc-0	aTc-10	aTc-100
Waterfoul *39*	-			
Waterfoul *40*	-			
Waterfoul *41*	-			
Waterfoul *42*	-			
Waterfoul *43*	-			
Waterfoul *44*	-			

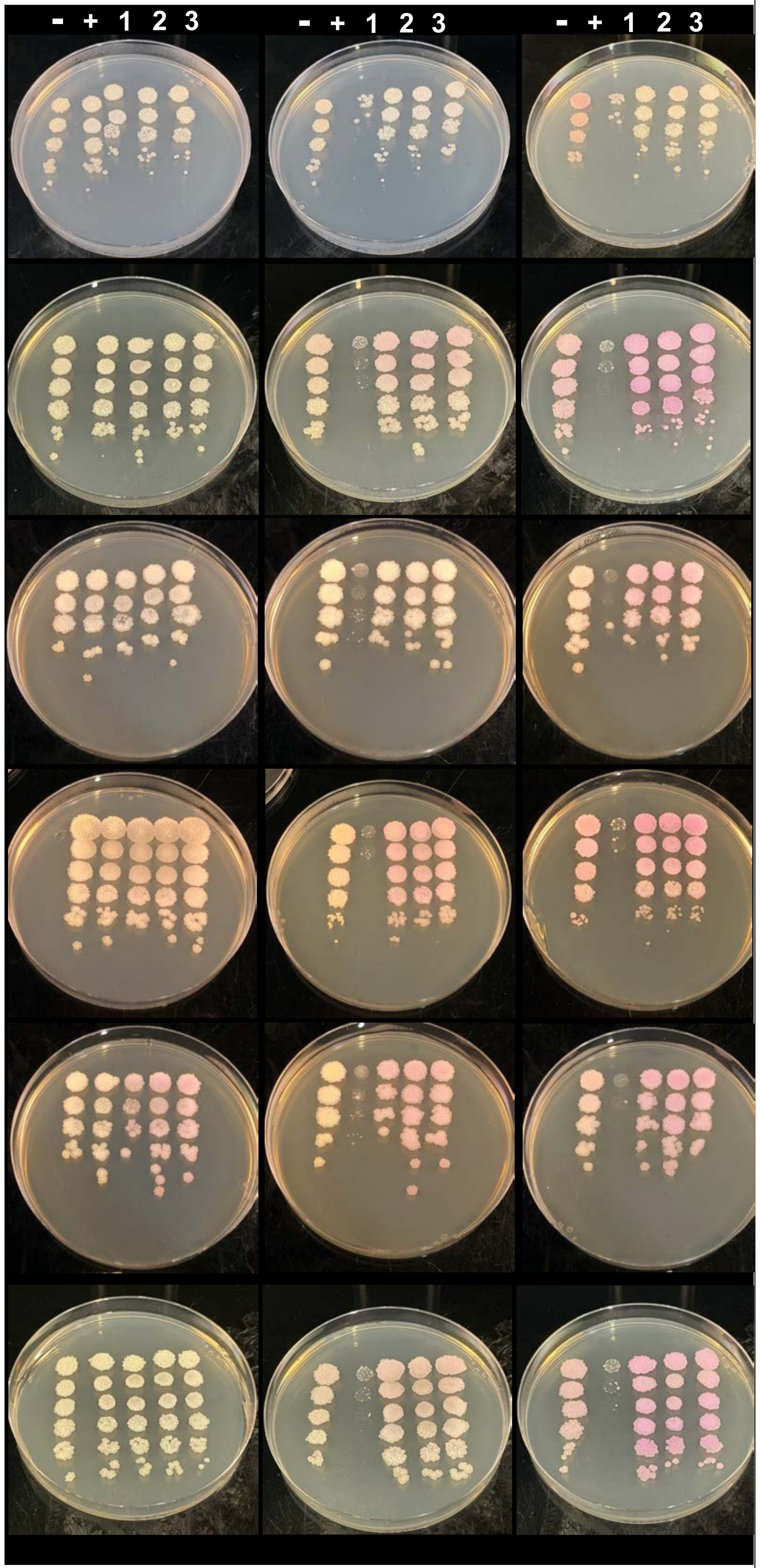

Gene	Score	aTc-0	aTc-10	aTc-100
Waterfoul *45*	-			
Waterfoul *46*	++			
Waterfoul *47*	+++			
Waterfoul *48*	++			
Waterfoul *49*	+++			
Waterfoul *50*	-			

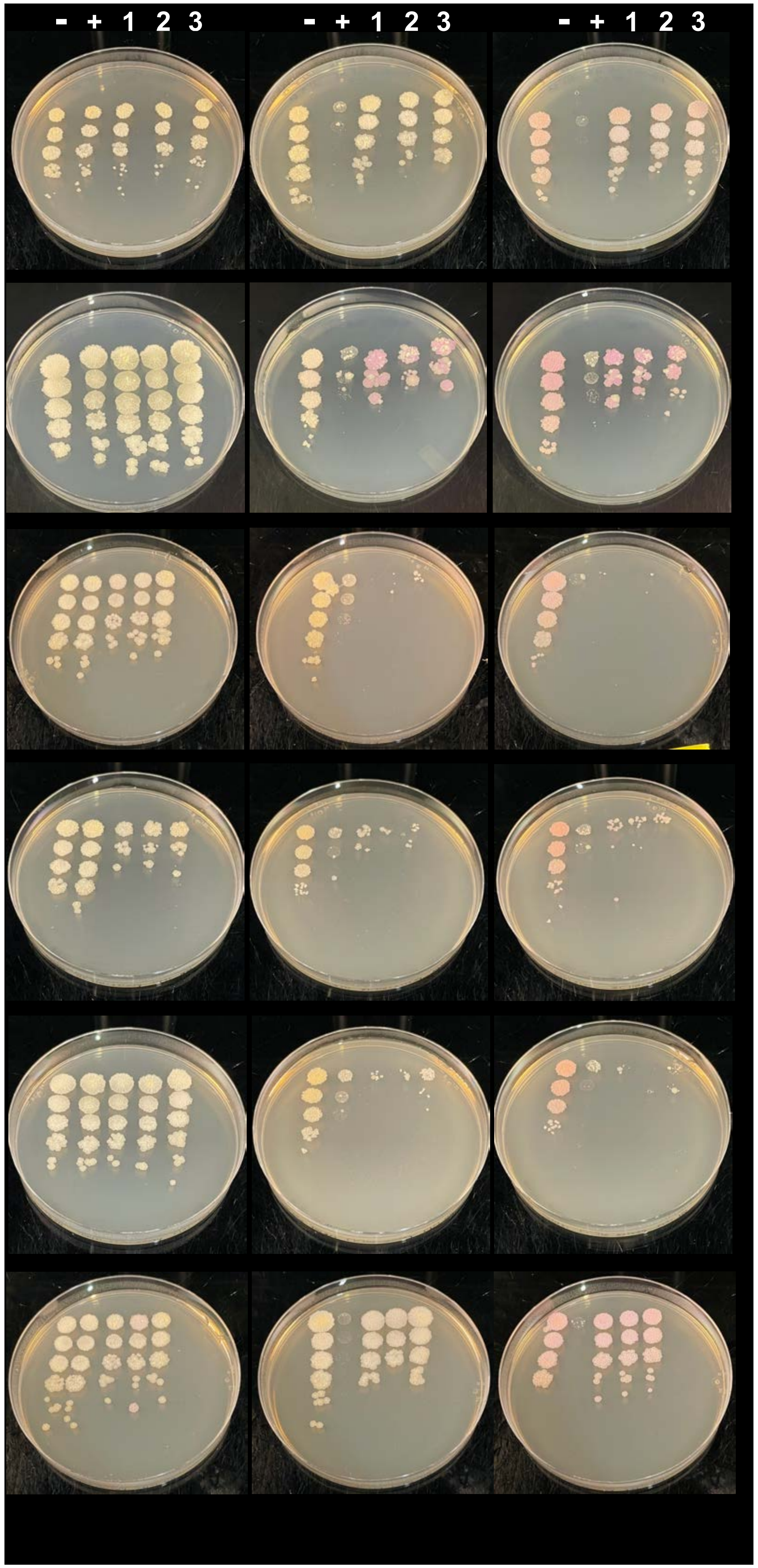

Gene	Score	aTc-0	aTc-10	aTc-100
Waterfoul *51*	-			
Waterfoul *52*	+++			
Waterfoul *53*	-			
Waterfoul *54*	-			
Waterfoul *55*	-			
Waterfoul *56*	-			

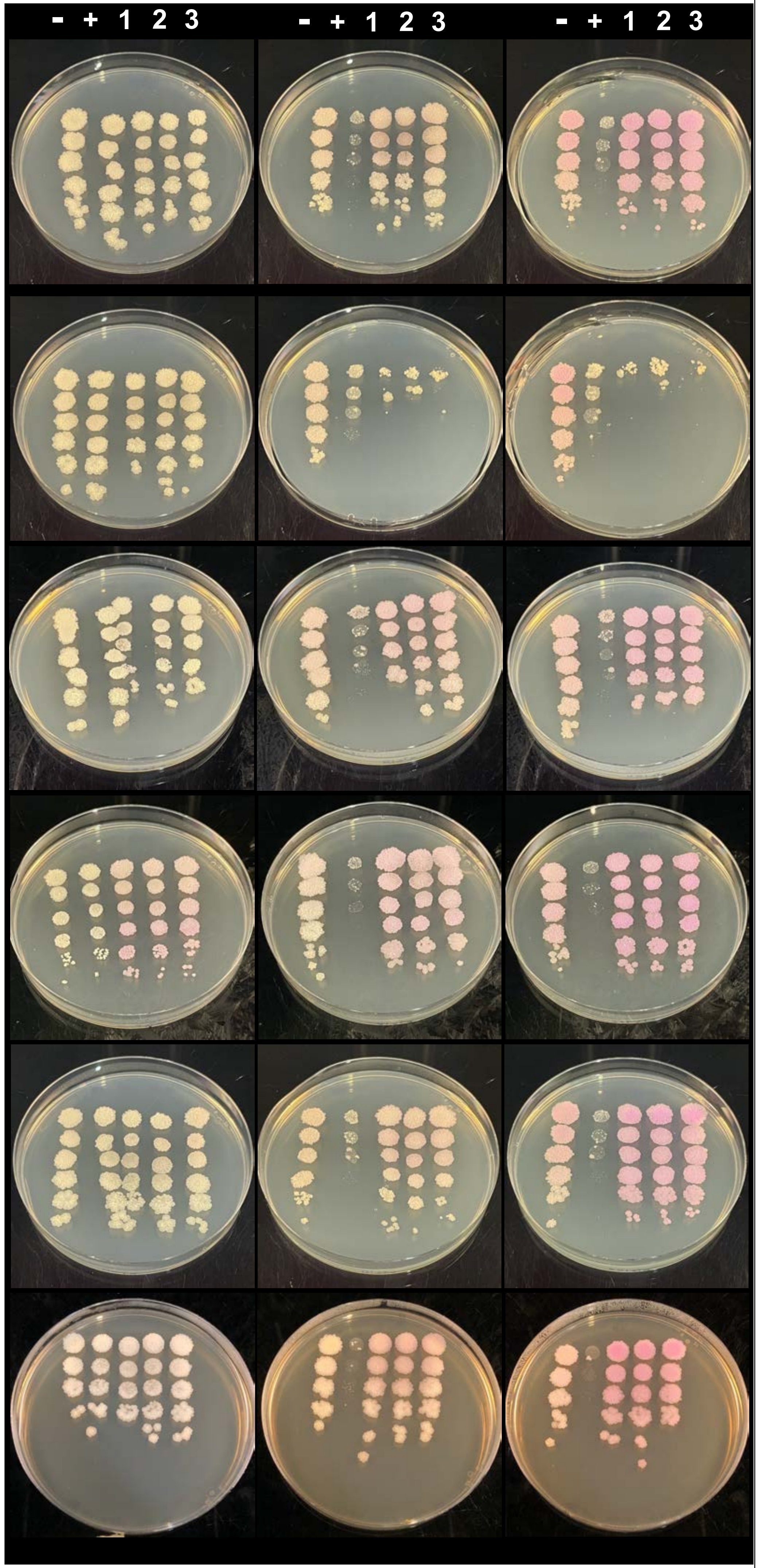

Gene	Score	aTc-0	aTc-10	aTc-100
Waterfoul *57*	++			
Waterfoul *58*	-			
Waterfoul *59*	-			
Waterfoul *60*	-			
Waterfoul *62*	+			
Waterfoul *61*	-			

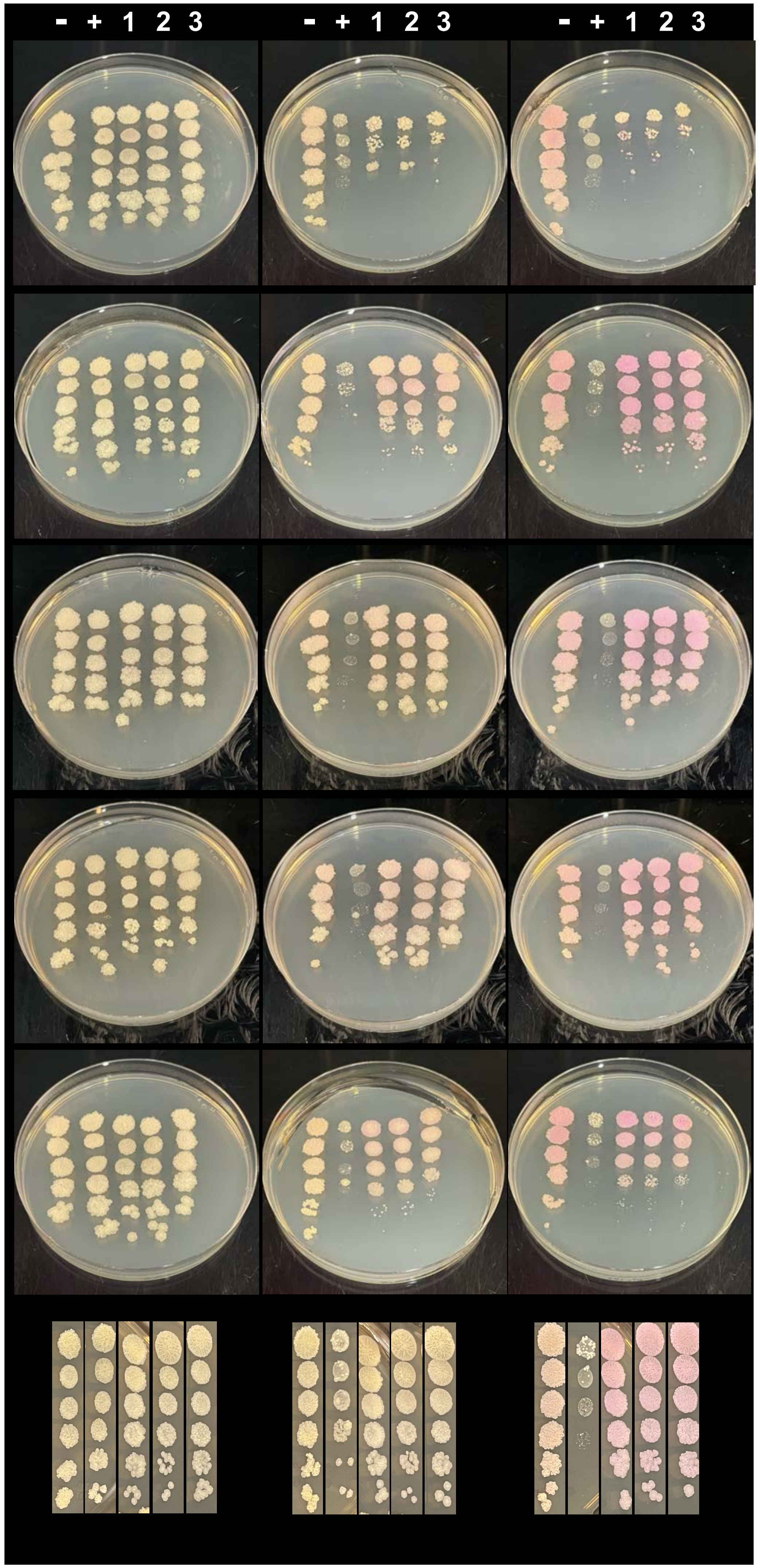

Gene	Score	aTc-0	aTc-10	aTc-100
Waterfoul *63*	+			
Waterfoul *64*	+			
Waterfoul *65*	+			
Waterfoul *66*	+			
Waterfoul *67*	-			
Waterfoul *68*	-			

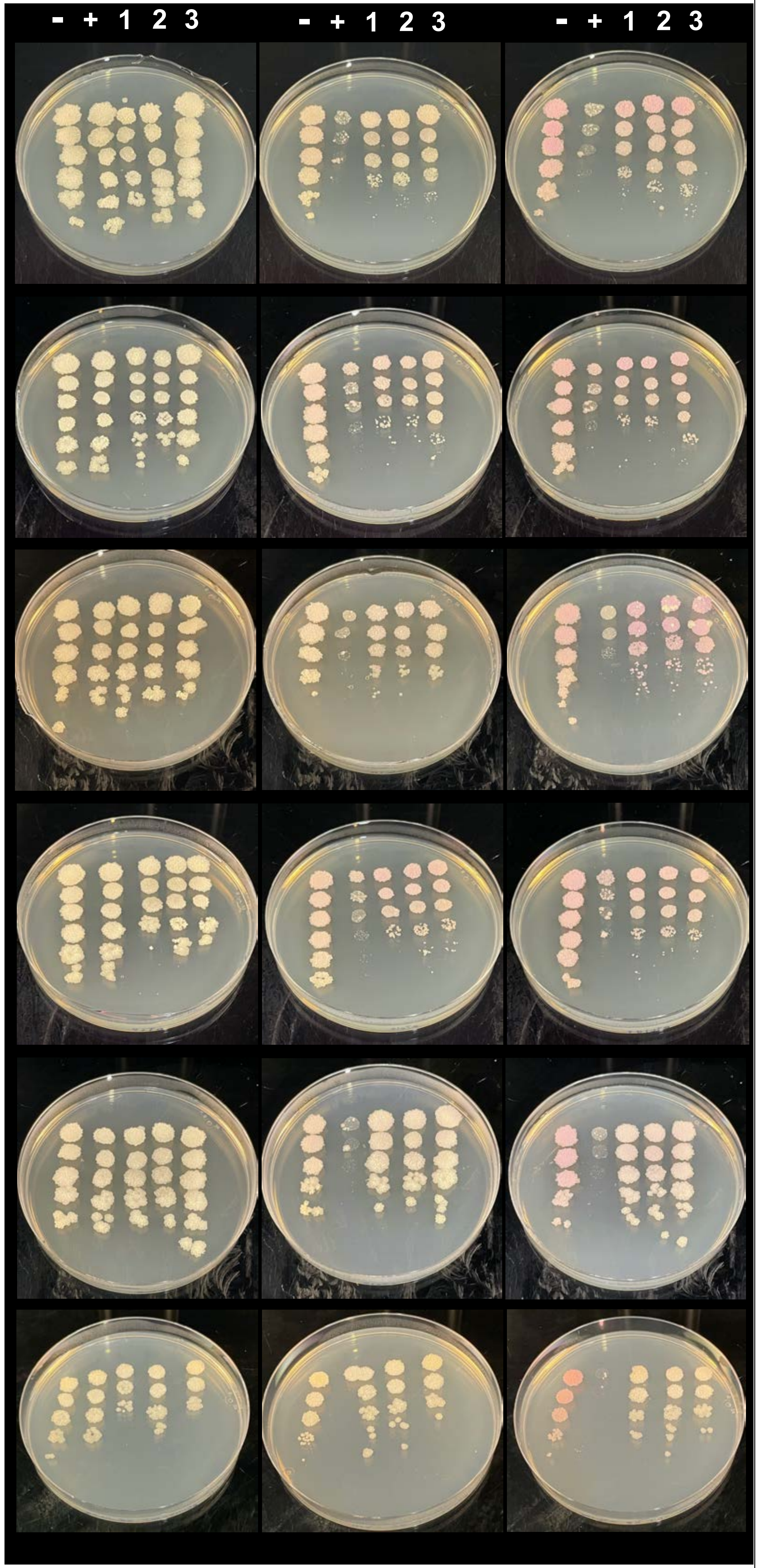

Gene	Score	aTc-0	aTc-10	aTc-100
Waterfoul *69*	-			
Waterfoul *70*	-			
Waterfoul *71*	+++			
Waterfoul *72*	-			
Waterfoul *73*	-			
Waterfoul *74*	-			

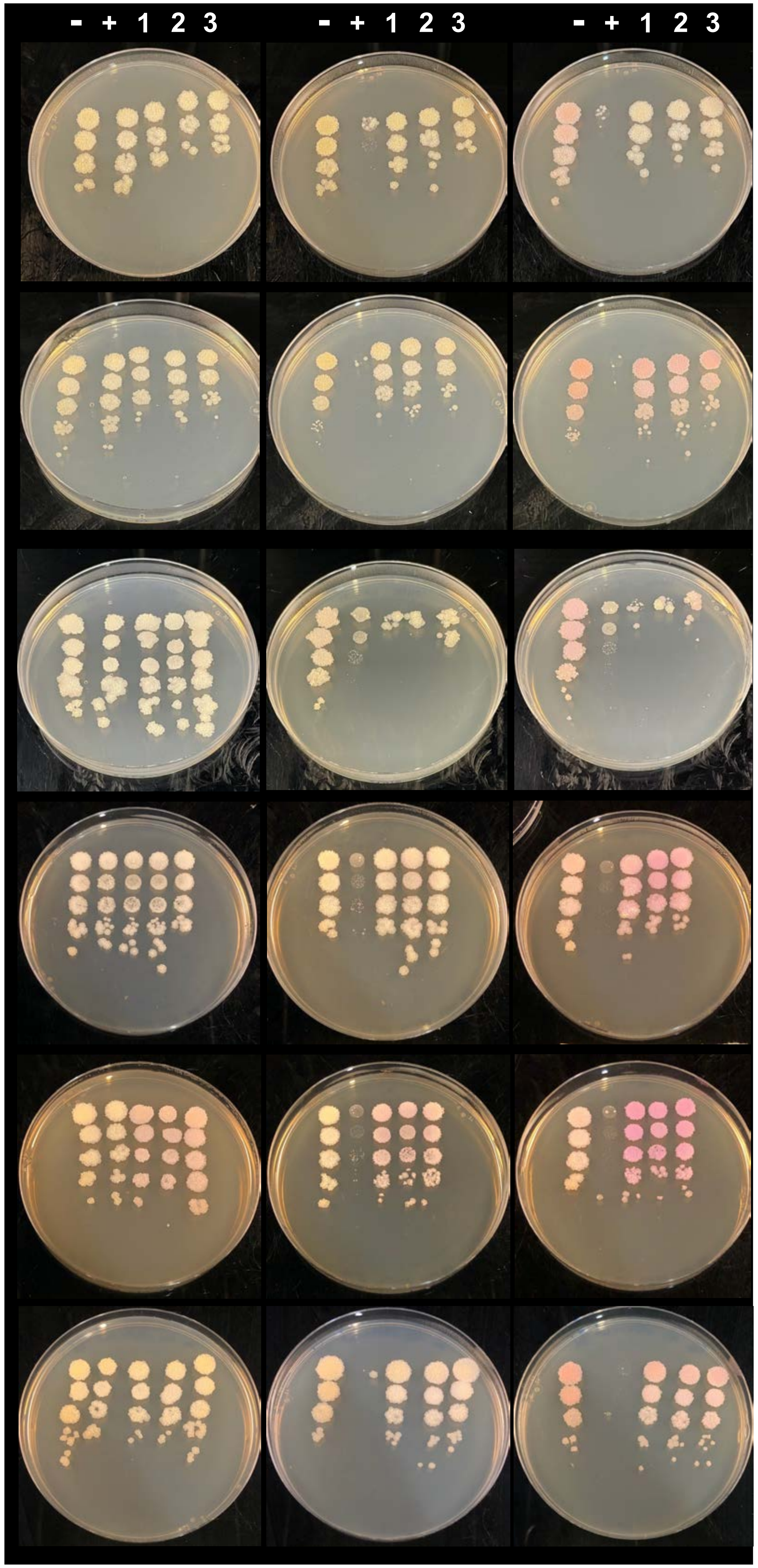

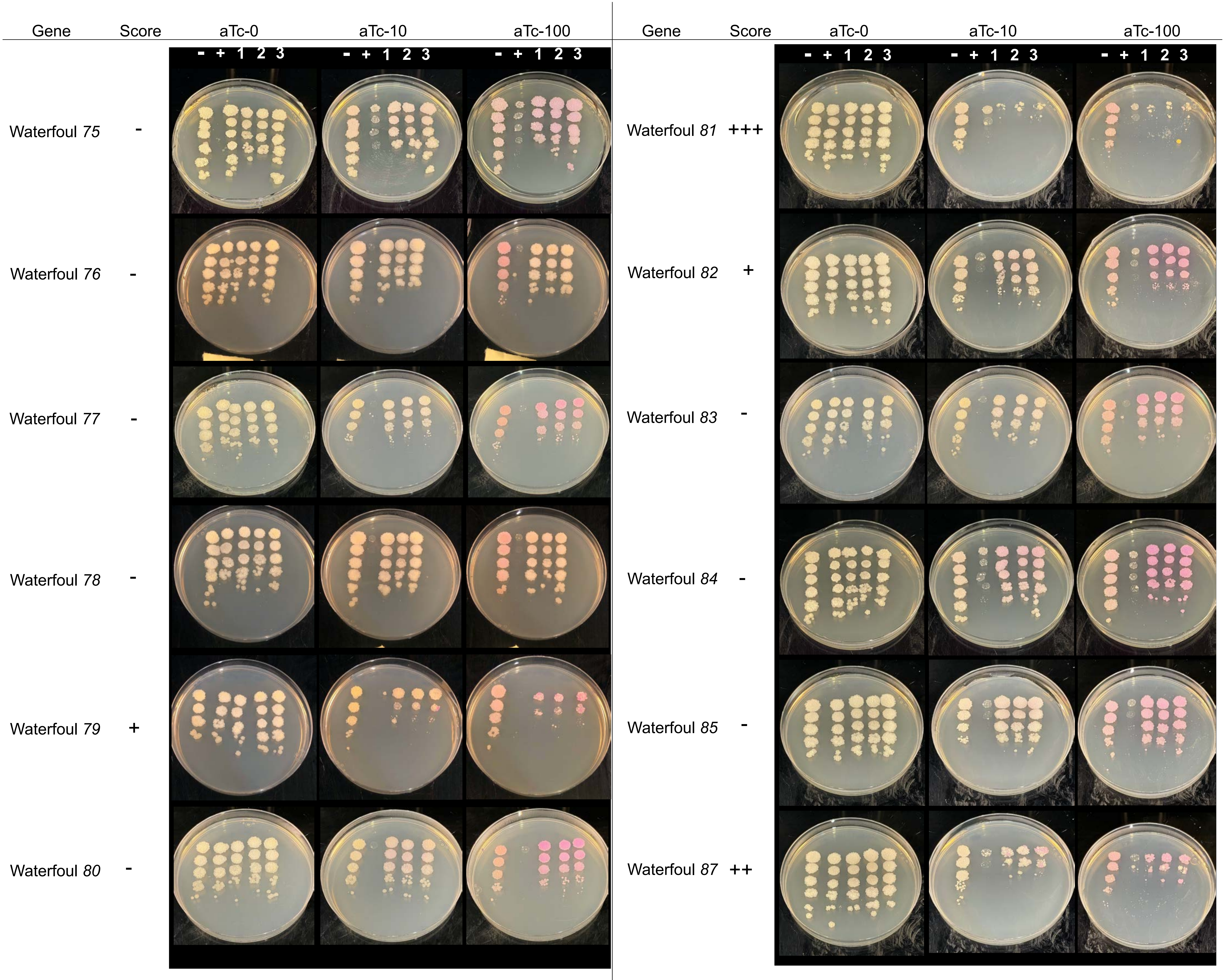
Gene
Score
aTc-0
aTc-10
aTc-100
- + 1 2 3
Waterfoul 75 -
Waterfoul 76 -
Waterfoul 77 -
Waterfoul 78 -
Waterfoul 79 +
Waterfoul 80 -
Waterfoul 81 +++
Waterfoul 82 +
Waterfoul 83 -
Waterfoul 84 -
Waterfoul 85 -
Waterfoul 87 ++

Gene	Score
Waterfoul *88*	+++
Waterfoul *89*	++
Waterfoul *90*	-
Waterfoul *91*	-
Waterfoul *92*	-
Waterfoul *93*	-

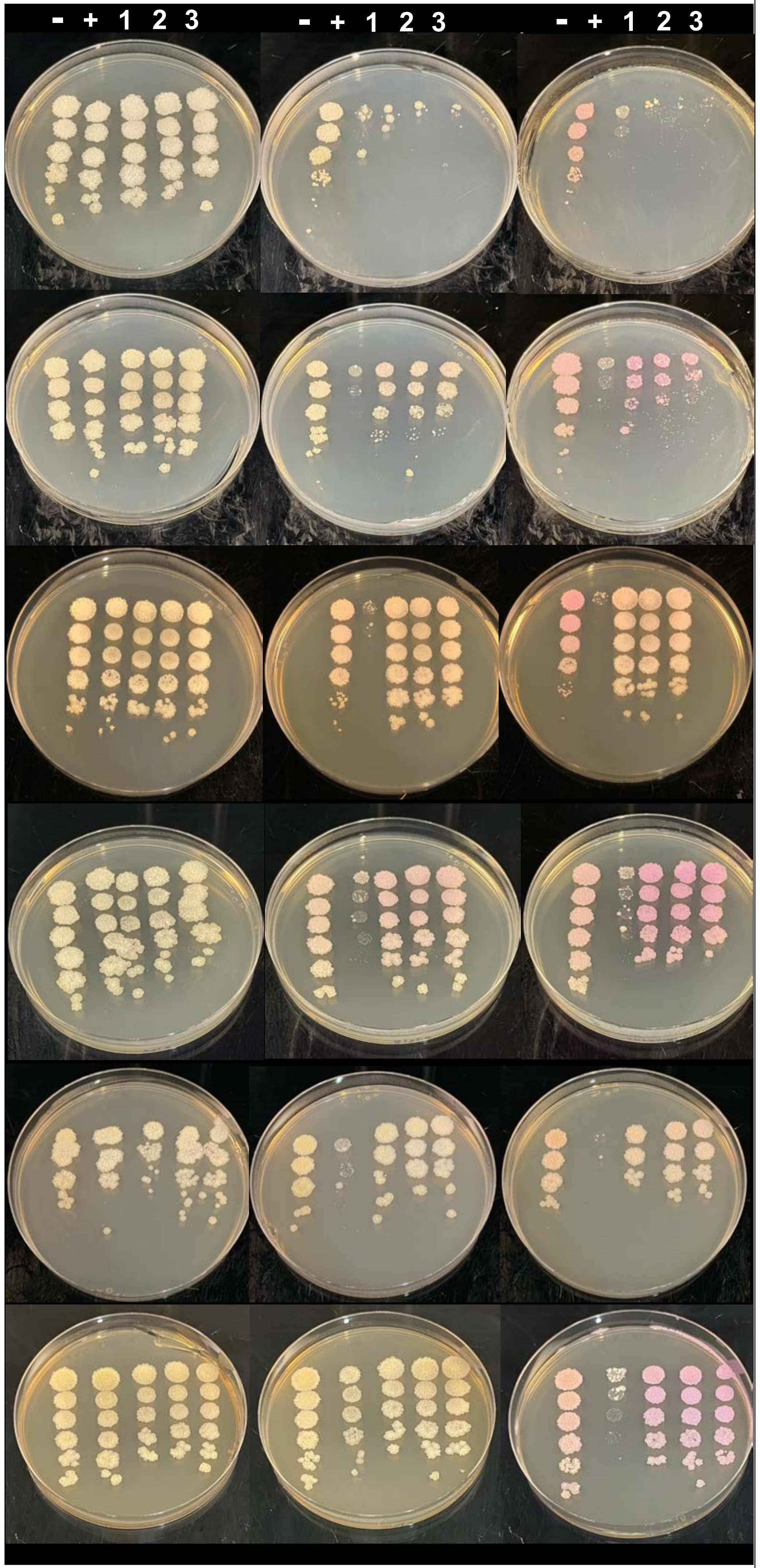

Gene	Score
Waterfoul *94*	-
Waterfoul *95*	-

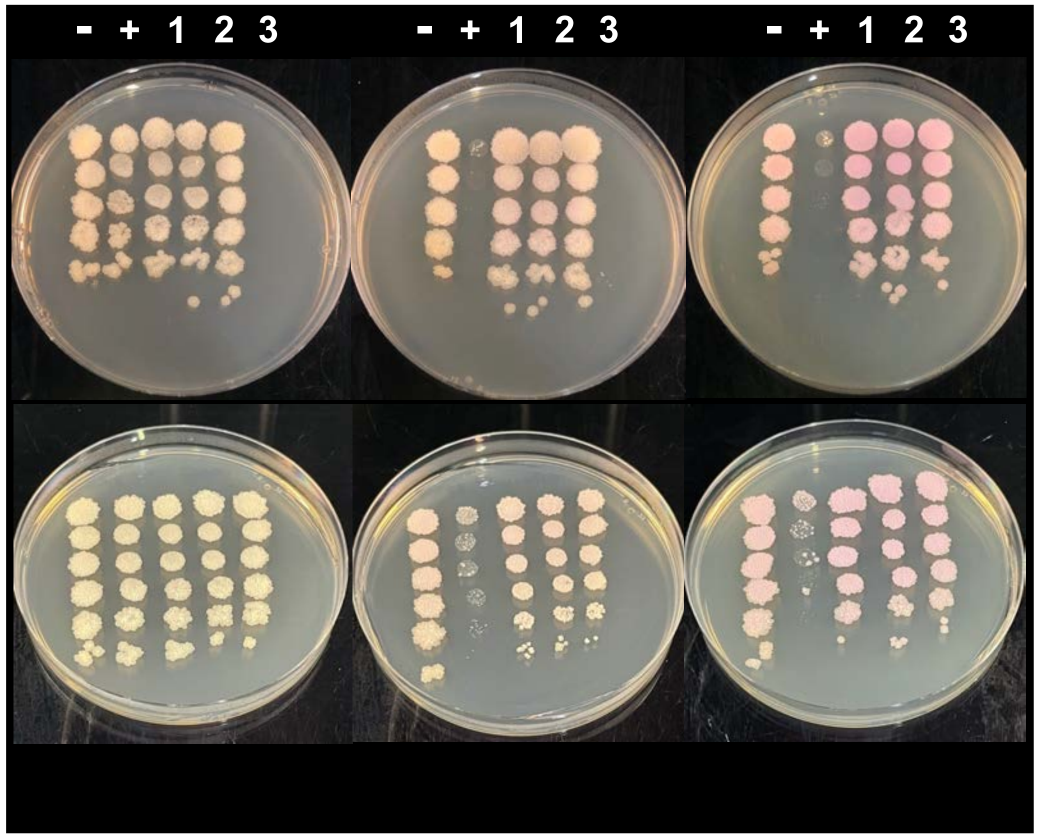

Supplement: jkac140_Supplemental_Figure_1 [file jkac140_supplemental_figure_1.pdf]
