# Supplementary material for: Systematic overexpression of genes encoded by mycobacteriophage Waterfoul reveals novel inhibitors of mycobacterial growth
Source: G3 (Bethesda). 2022 Jun 21;12(8):jkac140. doi: 10.1093/g3journal/jkac140 (PMC9339283; doi:10.1093/g3journal/jkac140)
Supplement: jkac140_Supplemental_Figure_1_Legend [file jkac140_supplemental_figure_1_legend.docx]

**Supplemental Figure 1:** Shown are the results of representative cytotoxicity assays for the 92 Waterfoul genes screened in this study. Each strain was spotted in triplicate alongside *M. smegmatis*/pExTra-Fruitloop52 (+) and pExTra-Fruitloop52I70S (-) control strains on 7H10 Kan supplemented with 0, 10, or 100 ng/ml aTc. In all experiments, 10^-1^ to 10^-5^ dilutions are shown; in some experiments the undiluted sample was also spotted. Plates were monitored over 5 days at 37 ºC, with day 5 results shown to best illustrate effects on colony color and size.
