# Supplementary material for: Systematic overexpression of genes encoded by mycobacteriophage Waterfoul reveals novel inhibitors of mycobacterial growth
Source: G3 (Bethesda). 2022 Jun 21;12(8):jkac140. doi: 10.1093/g3journal/jkac140 (PMC9339283; doi:10.1093/g3journal/jkac140)
Supplement: jkac140_Supplemental_Table_1 [file jkac140_supplemental_table_1.pdf]

Supplemental Table 1: DNA oligos used in this study

Oligo Name	Oligo Sequence (5' to 3')
oWaterfoul1_F	atgcggaggaatcacttccatATGGAGCAAGCCATCAACG
oWaterfoul1_R	tgcaggatccgactcgagtgtcgacTCACGCGTCACCGATGATG
oWaterfoul2_F	atgcggaggaatcacttccatATGATCGGCATTGTCGCGTC
oWaterfoul2_R	tgcaggatccgactcgagtgtcgacTCAAAGCTCTACCCCGCGC
oWaterfoul3_F	atgcggaggaatcacttccatATGACACCGACTGTCGGCCG
oWaterfoul3_R	tgcaggatccgactcgagtgtcgacTTACACGCGCGGCGGCCAG
oWaterfoul5_F	atgcggaggaatcacttccatATGGCTGCGAAGACAGAAGAC
oWaterfoul5_R	tgcaggatccgactcgagtgtcgacTCAGACATCCTCGCCGTCGAAC
oWaterfoul6_F	atgcggaggaatcacttccatATGTCTAACCCGCGGCTGTC
oWaterfoul6_R	tgcaggatccgactcgagtgtcgacTCATGCGAACATCGCGCC
oWaterfoul7_F	atgcggaggaatcacttccatATGATTCCGCTGTTCATCGAG
oWaterfoul7_R	tgcaggatccgactcgagtgtcgacTCACTCAGCCGTACTAGCG
oWaterfoul8_F	atgcggaggaatcacttccatATGCCGGAATTTCAGGGGAC
oWaterfoul8_R	tgcaggatccgactcgagtgtcgacTCACTGCAGATAATCCTCAGTC
oWaterfoul9_F	atgcggaggaatcacttccatATGACCGACATTGACGCGGTG
oWaterfoul9_R	tgcaggatccgactcgagtgtcgacTTACTCGGTGTCCTCCGCG
oWaterfoul10_F	atgcggaggaatcacttccatATGCCCGATGACGTGACCG
oWaterfoul10_R	tgcaggatccgactcgagtgtcgacTCAGCCGTTGCCGCGCAGTTC
oWaterfoul11_F	atgcggaggaatcacttccatATGGCCGACATCACGCGC
oWaterfoul11_R	tgcaggatccgactcgagtgtcgacTCACTCGCCGTCGTCTTCGG
oWaterfoul12_F	atgcggaggaatcacttccatATGCTGGCGACACTGGAC
oWaterfoul12_R	tgcaggatccgactcgagtgtcgacTCAGTACCGCTCGCTGCC
oWaterfoul13_F	atgcggaggaatcacttccatATGTTCCCGACGCCGCAC
oWaterfoul13_R	tgcaggatccgactcgagtgtcgacTCACTGAACGGGAATTCCAGC
oWaterfoul14_F	atgcggaggaatcacttccatATGGGCCGGCTGGAATTCC
oWaterfoul14_R	tgcaggatccgactcgagtgtcgacTCACGTCTGCGGCCCGTG
oWaterfoul15_F	atgcggaggaatcacttccatATGACGGTGCTCGTGCCG
oWaterfoul15_R	tgcaggatccgactcgagtgtcgacTCATCGGCGTGGCGGTTTCAG
oWaterfoul16_F	atgcggaggaatcacttccatATGACTGCTCCCGTAGAGCC
oWaterfoul16_R	tgcaggatccgactcgagtgtcgacTCAGACGGCGATGACGCCG
oWaterfoul17_F	atgcggaggaatcacttccatATGCCCAAGACCAAGAACGAAG
oWaterfoul17_R	tgcaggatccgactcgagtgtcgacTCACCTGCCGCGCCGCCGCGCAG
oWaterfoul18_F	atgcggaggaatcacttccatATGCCCAAGACCAAGAACG
oWaterfoul18_R	tgcaggatccgactcgagtgtcgacTCACTTGTTCTTGCGTTCCAAC
oWaterfoul19_F	atgcggaggaatcacttccatATGGCTGAAGCGAAGTATTACC
oWaterfoul19_R	tgcaggatccgactcgagtgtcgacTCATCGCAGATGTGTTGTTCGG

oWaterfoul20_F	atgcggaggaatcacttccatATGTCTGGGCTTCACGATGATTG
oWaterfoul20_R	tgcaggatccgactcgagtgtcgacTCACGCCCACGCCATCTCG
oWaterfoul21_F	atgcggaggaatcacttccatATGATTTTGACTGTTCCCAACC
oWaterfoul21_R	tgcaggatccgactcgagtgtcgacTCAGAACAGGTCTCCTGATCCG
oWaterfoul22_F	atgcggaggaatcacttccatATGGAACTGCAGCACATGC
oWaterfoul22_R	tgcaggatccgactcgagtgtcgacTCATTCGGTGGACTCCTCG
oWaterfoul23_F	atgcggaggaatcacttccatATGACGAAGATCGCAACGAC
oWaterfoul23_R	tgcaggatccgactcgagtgtcgacTCAGAGCCCCTGCGGAAC
oWaterfoul24_F	atgcggaggaatcacttccatATGGGCGCCTCGAAGCAAT
oWaterfoul24_R	tgcaggatccgactcgagtgtcgacTCATACGCTCTTGGCATGCG
oWaterfoul25_F	atgcggaggaatcacttccatATGCCAAGAGCGTATGACC
oWaterfoul25_R	tgcaggatccgactcgagtgtcgacTCACAGGTCCATCATTTCAAAGTTG
oWaterfoul26_F	atgcggaggaatcacttccatATGCTGGAAATCAGAAAGCAACTC
oWaterfoul26_R	tgcaggatccgactcgagtgtcgacTCAAGAGACCACGCCACCG
oWaterfoul27_F	atgcggaggaatcacttccatATGGCGTGGTCTCTTGACC
oWaterfoul27_R	tgcaggatccgactcgagtgtcgacTTACTGGTATGCGACGCACCA
oWaterfoul28_F	atgcggaggaatcacttccatATGTCGGAATATCAAGCCGCG
oWaterfoul28_R	tgcaggatccgactcgagtgtcgacCTACTCGCCGCGGTACTTGAA
oWaterfoul29_F	atgcggaggaatcacttccatATGGACGTGACACGCGG
oWaterfoul29_R	tgcaggatccgactcgagtgtcgacTCACGCAGTCACACCCTTG
oWaterfoul30_F	atgcggaggaatcacttccatATGAGCAAGCCAATGTTGTTGACC
oWaterfoul30_R	tgcaggatccgactcgagtgtcgacTCATGCCGCCATCGCCCG
oWaterfoul31_F	atgcggaggaatcacttccatATGACCTCGGGCGCTGATAG
oWaterfoul31_R	tgcaggatccgactcgagtgtcgacTCACGCGGCAACCTGCC
oWaterfoul32_F	atgcggaggaatcacttccatATGACTGGCAGTGCGGTG
oWaterfoul32_R	tgcaggatccgactcgagtgtcgacTCAGCCAGATTCACGGGC
oWaterfoul33_F	atgcggaggaatcacttccatATGAATCTGGCTGAACATCTCGG
oWaterfoul33_R	tgcaggatccgactcgagtgtcgacTCAGACAGCGACGACACGC
oWaterfoul34_F	atgcggaggaatcacttccatATGTCGTCGCTGTCTGAGC
oWaterfoul34_R	tgcaggatccgactcgagtgtcgacCTACACGATCCCCATGCGC
oWaterfoul35_F	atgcggaggaatcacttccatATGCGTGGCGAACATGCAATG
oWaterfoul35_R	tgcaggatccgactcgagtgtcgacTCACAGGTCGACCGTCCGC
oWaterfoul36_F	atgcggaggaatcacttccatATGAGGCTGTCACCGAAGC
oWaterfoul36_R	tgcaggatccgactcgagtgtcgacTCAGCCGCGCGCATGGTC
oWaterfoul37_F	atgcggaggaatcacttccatATGCGCGCGGCTGATCG
oWaterfoul37_R	tgcaggatccgactcgagtgtcgacTCAGCGACGAACGACGTCAG
oWaterfoul38_F	atgcggaggaatcacttccatATGACTACGGGAACGATCGACT
oWaterfoul38_R	tgcaggatccgactcgagtgtcgacTCACCGGCGATCTTTTTTGCC
oWaterfoul39_F	atgcggaggaatcacttccatATGCGCGCCATCATTTACAACC

oWaterfoul39_R	tgcaggatccgactcgagtgtcgacTCACGCATCAGCGTGCGGG
oWaterfoul40_F	atgcggaggaatcacttccatATGACAGGGCAGCGGCTC
oWaterfoul40_R	tgcaggatccgactcgagtgtcgacCTACGCGGCGGGTTGCTT
oWaterfoul41_F	atgcggaggaatcacttccatATGACGCTTGACCTCATCGC
oWaterfoul41_R	tgcaggatccgactcgagtgtcgacTTAAAGCGCGGGCGCGGGCTCGT
oWaterfoul42_F	atgcggaggaatcacttccatATGACCATGAGTAGCGACTAC
oWaterfoul42_R	tgcaggatccgactcgagtgtcgacTCACAGAGGATCTGCGTCACC
oWaterfoul43_F	atgcggaggaatcacttccatATGTCCGCTTTTCCGGTCATC
oWaterfoul43_R	tgcaggatccgactcgagtgtcgacTCAACCTGCCCGTTTCGTC
oWaterfoul44_F	atgcggaggaatcacttccatATGTTGGGACAACAACACCGG
oWaterfoul44_R	tgcaggatccgactcgagtgtcgacTTATCCGCAGCGGTCCATGTC
oWaterfoul45_F	atgcggaggaatcacttccatATGTCGAAACCAAGTATAAACCACGA
oWaterfoul45_R	tgcaggatccgactcgagtgtcgacTCACAGCAGACCTACGGACA
oWaterfoul46_F	atgcggaggaatcacttccatATGAGCACCATCACCGAAATTCG
oWaterfoul46_R	tgcaggatccgactcgagtgtcgacTCAGCCGACCGCTGCGCTGTT
oWaterfoul47_F	atgcggaggaatcacttccatATGACCAGCCACGCAATGC
oWaterfoul47_R	tgcaggatccgactcgagtgtcgacCTACGGTGCGCCAGCGTC
oWaterfoul48_F	atgcggaggaatcacttccatATGTTGACCATCAACACCGCAGAC
oWaterfoul48_R	tgcaggatccgactcgagtgtcgacCTACTTGCCGTCGTCGCC
oWaterfoul49_F	atgcggaggaatcacttccatATGAAGAAGCGCATTGCAATCGT
oWaterfoul49_R	tgcaggatccgactcgagtgtcgacTCAGTCCCGCGGGGTGAC
oWaterfoul50_F	atgcggaggaatcacttccatATGAGCAGCGGTGAACGC
oWaterfoul50_R	tgcaggatccgactcgagtgtcgacTCATTGGTTCACTTTCTCGTTGTAG
oWaterfoul51_F	atgcggaggaatcacttccatATGACCGACATCGCGACAGT
oWaterfoul51_R	tgcaggatccgactcgagtgtcgacTCACTCTGCAAGCTCGATCC
oWaterfoul52_F	atgcggaggaatcacttccatATGACTGAGTTTGTGCTCGCC
oWaterfoul52_R	tgcaggatccgactcgagtgtcgacTTACTTGAACCGGCCGACGAT
oWaterfoul53_F	atgcggaggaatcacttccatATGAGGGTCACACGGCGC
oWaterfoul53_R	tgcaggatccgactcgagtgtcgacCTACTCGGTGCTCATGTGGTCG
oWaterfoul54_F	atgcggaggaatcacttccatATGAAGCGAAACAAAGAGAAGC
oWaterfoul54_R	tgcaggatccgactcgagtgtcgacTCATCTCGCCCACAACTTCG
oWaterfoul55_F	atgcggaggaatcacttccatATGAGCCAGGAATTCGTGATGAT
oWaterfoul55_R	tgcaggatccgactcgagtgtcgacTCACTGCTCCGCCCCCAC
oWaterfoul56_F	atgcggaggaatcacttccatATGAAAGCCACCTTGCGGC
oWaterfoul56_R	tgcaggatccgactcgagtgtcgacTCATGAAAGCATCCCCTTAATCCA
oWaterfoul57_F	atgcggaggaatcacttccatATGACGAACGGGACGGCC
oWaterfoul57_R	tgcaggatccgactcgagtgtcgacTCACGACACACCGCCACG
oWaterfoul58_F	atgcggaggaatcacttccatATGACGGCCCCGAACGTG
oWaterfoul58_R	tgcaggatccgactcgagtgtcgacTCACGACTCCACCTCACGGT

oWaterfoul59_F	atgcggaggaatcacttccatATGAGCGCGGCAACGTTC
oWaterfoul59_R	tgcaggatccgactcgagtgtcgacTTACGGGCAGTCGCCGGC
oWaterfoul60_F	atgcggaggaatcacttccatATGGCCGATCACTACTGCAC
oWaterfoul60_R	tgcaggatccgactcgagtgtcgacTTATGTGCCGTCGTAATAGTCGGG
oWaterfoul61_F	atgcggaggaatcacttccatATGACGAACGCATCGAAGCAATTC
oWaterfoul61_R	tgcaggatccgactcgagtgtcgacTTACTGCTTGAGCATCCCGAGTTC
oWaterfoul62_F	atgcggaggaatcacttccatATGATCACCATTTACACAACCGGC
oWaterfoul62_R	tgcaggatccgactcgagtgtcgacTCACGGGTTGATCCCCCGC
oWaterfoul63_F	atgcggaggaatcacttccatATGACCGTGCGCAGAGGAA
oWaterfoul63_R	tgcaggatccgactcgagtgtcgacTCATTTCAGGTGACCCCAGTCG
oWaterfoul64_F	atgcggaggaatcacttccatATGAGCGACCGCGCAACAT
oWaterfoul64_R	tgcaggatccgactcgagtgtcgacTCAGAGTGTGTGCGAACCTG
oWaterfoul65_F	atgcggaggaatcacttccatATGACCCTGACCATCCCTG
oWaterfoul65_R	tgcaggatccgactcgagtgtcgacTCACTCGTCTTCGCTTTCTG
oWaterfoul66_F	atgcggaggaatcacttccatATGACCGGCCAGGAACGG
oWaterfoul66_R	tgcaggatccgactcgagtgtcgacTCAGCTCGCCCACTCGAC
oWaterfoul67_F	atgcggaggaatcacttccatATGGGCCGCACCATGCGA
oWaterfoul67_R	tgcaggatccgactcgagtgtcgacTCAGTGAGTCACCTTCTCGCC
oWaterfoul68_F	atgcggaggaatcacttccatATGCCCGGCACATGCGG
oWaterfoul68_R	tgcaggatccgactcgagtgtcgacTCAACACGCTGCCGGCG
oWaterfoul69_F	atgcggaggaatcacttccatATGCAGATTTGCGAACTGTTCAC
oWaterfoul69_R	tgcaggatccgactcgagtgtcgacTTACGCGTGGACGTTGCCG
oWaterfoul70_F	atgcggaggaatcacttccatATGCTGAAGAACATCCTGAAGGC
oWaterfoul70_R	tgcaggatccgactcgagtgtcgacTCATGCCGGCTGCGCTTC
oWaterfoul71_F	atgcggaggaatcacttccatATGACCGCACACACCGATG
oWaterfoul71_R	tgcaggatccgactcgagtgtcgacTCATGCGTTCACTTCCTCACG
oWaterfoul72_F	atgcggaggaatcacttccatATGACTGTCGGCGAACTAATCG
oWaterfoul72_R	tgcaggatccgactcgagtgtcgacTCACAGTTCCCTCACATCCGAC
oWaterfoul73_F	atgcggaggaatcacttccatATGACCGCCCCGAACTATC
oWaterfoul73_R	tgcaggatccgactcgagtgtcgacTCATTGCGCGCACCACAC
oWaterfoul74_F	atgcggaggaatcacttccatATGACCGCGCCAAGCGAG
oWaterfoul74_R	tgcaggatccgactcgagtgtcgacCTATCTCTGTCGTGTCACGTTGT
oWaterfoul75_F	atgcggaggaatcacttccatATGAGCGTCGCTGAGCAGT
oWaterfoul75_R	tgcaggatccgactcgagtgtcgacTCACCACAGGGCGCCTTG
oWaterfoul76_F	atgcggaggaatcacttccatATGCTGAGCGTTGAACCCG
oWaterfoul76_R	tgcaggatccgactcgagtgtcgacTCACTTGGGTTCTGATCGGTCG
oWaterfoul77_F	atgcggaggaatcacttccatATGGGGAAACACAGCGCG
oWaterfoul77_R	tgcaggatccgactcgagtgtcgacTTACCCGTTGTCGGCCTG
oWaterfoul78_F	atgcggaggaatcacttccatATGCGCAAAATACCGAGTCTGTT

oWaterfoul78_R	tgcaggatccgactcgagtgtcgacTCACTGCCGGGCCGCCTT
oWaterfoul79_F	atgcggaggaatcacttccatATGACCTACATCGAAGTGCGC
oWaterfoul79_R	tgcaggatccgactcgagtgtcgacTCACCAGCTGCCGTCCTC
oWaterfoul80_F	atgcggaggaatcacttccatATGAGCGCCACGCAAGGC
oWaterfoul80_R	tgcaggatccgactcgagtgtcgacTTACGGTTGCAGCTCCTTGAA
oWaterfoul81_F	atgcggaggaatcacttccatATGGGCAAGCACAGCATGG
oWaterfoul81_R	tgcaggatccgactcgagtgtcgacTCAGCCATGCCGCAACAC
oWaterfoul82_F	atgcggaggaatcacttccatATGGCTGACAAGAACGACGAA
oWaterfoul82_R	tgcaggatccgactcgagtgtcgacTCACGCCCATTGCGTCAC
oWaterfoul83_F	atgcggaggaatcacttccatATGGGCGTGACACGCGCAG
oWaterfoul83_R	tgcaggatccgactcgagtgtcgacTCATTTCGGTTTCCTCAATCCCAT
oWaterfoul84_F	atgcggaggaatcacttccatATGACCGACACGCAACATGC
oWaterfoul84_R	tgcaggatccgactcgagtgtcgacTCAGGCAGTGCCGAACAGTC
oWaterfoul85_F	atgcggaggaatcacttccatATGAAGAACAACACCGCTCAGC
oWaterfoul85_R	tgcaggatccgactcgagtgtcgacTTAGATCCGGGCGGCGCG
oWaterfoul86_F	atgcggaggaatcacttccatATGGCGAAATGGACACACGA
oWaterfoul86_R	tgcaggatccgactcgagtgtcgacCTACTCTCCTTCCTGGTAGATCC
oWaterfoul87_F	atgcggaggaatcacttccatATGATGAGTAAGCAAACCCGCG
oWaterfoul87_R	tgcaggatccgactcgagtgtcgacTTACCCGGCGCTTTGCATG
oWaterfoul88_F	atgcggaggaatcacttccatATGACGAACTGGACCAAGACC
oWaterfoul88_R	tgcaggatccgactcgagtgtcgacTTACTTCTTCGGCAGCTTGTGC
oWaterfoul89_F	atgcggaggaatcacttccatATGTTCAAGATGATCGTTCAACTTC
oWaterfoul89_R	tgcaggatccgactcgagtgtcgacTCAGGCGGCCGGCACGC
oWaterfoul90_F	atgcggaggaatcacttccatATGCCGCTCATCATCTCCC
oWaterfoul90_R	tgcaggatccgactcgagtgtcgacTCAGATGTGGTTGCGGCC
oWaterfoul91_F	atgcggaggaatcacttccatATGACCACGATCGACACTCGC
oWaterfoul91_R	tgcaggatccgactcgagtgtcgacTCAGGCGCCTAGCGCTGG
oWaterfoul92_F	atgcggaggaatcacttccatATGATCGCCACCGCAACC
oWaterfoul92_R	tgcaggatccgactcgagtgtcgacTCAGGCCATGATGAATCCGAC
oWaterfoul93_F	atgcggaggaatcacttccatATGAACCGCGAACCAGAGC
oWaterfoul93_R	tgcaggatccgactcgagtgtcgacCTACGTATGCGCGGCCGG
oWaterfoul94_F	atgcggaggaatcacttccatATGGCCGGCCACGAACAC
oWaterfoul94_R	tgcaggatccgactcgagtgtcgacTCATGGCCTGTATGGCTCG
oWaterfoul95_F	atgcggaggaatcacttccatATGACAGTCACGCGCAGC
oWaterfoul95_R	tgcaggatccgactcgagtgtcgacTTACGGCTTCCATTCGCGG
oDH117	atgGAAGTGATTCCTCCGCATG
oDH152	catgcggaggaatcacttccatatgtagtgaGTCGACACTCGAGTCGGATCCTGCAGGAAGCTTGATCCGATAACACAGGAACAGATCTatggtctcgaagggcgaggag
oDH153	aagctatcagttaactaaaacg
oDH154	tcgttttagttaactgatagcttttacttgtacagctcgtccatgc

pExTra_F	GTCGACACTCGAGTCGGATCCTG
pExTra_R	ATGGAAGTGATTCCTCCGCATGC
pExTra_seqF	GTACCCGTGTGTACGACCAGC
pExTra_uniR	CCCTTCGAGACCATAGATCTGTTCC
oWaterfoul8_internalseq1	GCAAGCCAGGCCGGCCACGG
oWaterfoul8_internalseq2	GACGCGGTGGCTGATTGG
oWaterfoul19_internalseq1	CAGACGTCGGGCAAGGCGTTC
oWaterfoul19_internalseq2	TTCGTCGCTGACAACGCATC
oWaterfoul19_internalseq3	TGGCGTAGCCCGACAGTG
oWaterfoul19_internalseq4	TCTTCACGGAGTTCAGTGAGCC
oWaterfoul21_internalseq	CTGACGGTGCTCATGGC
oWaterfoul25_internalseq1	ACACGATCGTGACGTCGAACG
oWaterfoul25_internalseq2	ACAGCGGCGGTGACTGCTCG
oWaterfoul29_internalseq1	CATCGTGTATTTCGTGTGG
oWaterfoul65_internalseq1	ACGCTGACGCAGCCACCGAC
oWaterfoul65_internalseq2	ACGAACCGTTGATGCCGG
oWaterfoul66_internalseq1	CAAGTTCGTCACTGCCGCG
oFruitloop52_F	atgcggaggaatcacttccatATGCTAACCCCAGATTCTAAACCCG
oFruiitloop52_R	tgcaggatccgactcgagtgtcgacTCATGCTGCGTCTCCCTCG
